# Nitrous oxide for late-life depression with inadequate antidepressant response: a randomised controlled trial

**DOI:** 10.1016/j.eclinm.2026.103860

**Published:** 2026-04-02

**Authors:** Antony Salamé, Samuel Bulteau, Gabriel Robert, Anaïs Vandevelde, Valérie Gissot, Marie-Sara Agier, Quentin Gallet, Jacques-Alexis Nkodo, Victoire Leroy, Paul Brunault, Hélène Bourgoin, Nicolas Arlicot, Fabien Espitalier, Amélie Le Gouge, Wissam El-Hage, Vincent Camus, Pierre Poupin, Thomas Desmidt

**Affiliations:** a CHU de Tours, Tours, France; b Université de Tours, INSERM, Imaging Brain & Neuropsychiatry iBraiN U1253, 37032, Tours, France; c CHU de Tours, INSERM, CIC1415, 37000, Tours, France; d CHU de Nantes, Addictology and Liaison Psychiatry Department, 44000, Nantes, France; e Empenn U1228, INSERM, INRIA, Université de Rennes, UMR CNRS 6074 IRISA, Campus de Beaulieu, 35042, Rennes, Cedex, France; f CIC 1414, CHU de Rennes, INSERM, Rennes, France; g CHU de Angers, Angers, France; h INSERM, SPHERE, U1246, Nantes University, Tours University, France

**Keywords:** Nitrous oxide, Late-life depression, Inadequate antidepressant response

## Abstract

**Background:**

Late-life depression with inadequate antidepressant response is a pressing clinical issue with limited proven therapies. Nitrous oxide (N_2_O) may offer a new therapeutic avenue with rapid and safe antidepressant effects in late-life depression with inadequate antidepressant response.

**Methods:**

We conducted a double-blind, randomised, placebo-controlled, single-dose trial (November 2021–December 2024) in three old-age psychiatry departments at French university hospitals. Eligible participants were aged 60–90 years with a current major depressive episode, a Montgomery-Åsberg Depression Rating Scale (MADRS) score >20 and documented non-response to ≥1 adequate antidepressant trial. Participants were randomly assigned (1:1) to a single 60-min inhalation of either 50% N_2_O/50% O_2_ (N_2_O group) or medical air (placebo group). The primary outcome was change in MADRS score from baseline to 2 weeks, analysed under the intention-to-treat principle with a linear mixed-effects model using all available data. Secondary outcomes included clinician- and self-rated depressive symptom scales and safety. The study is complete and registered in ClinicalTrials.gov (NCT05007028).

**Findings:**

Among the 60 participants enrolled in the study, those who received N_2_O experienced a significant and sustained reduction in depressive symptoms compared to the placebo group. The antidepressant effect was rapid, with significant improvements noted as early as 24 h and at one week. At the two-week endpoint, the N2O group showed a significantly greater reduction in MADRS scores (−6.2 points; 95% CI: −9.1 to −3.4; P < 0.001). These positive findings were supported by both clinician- and self-rated questionnaires, and all reported adverse events were mild and transient.

**Interpretation:**

While further studies are needed to confirm its long-term efficacy and safety, our findings suggest that N_2_O offers a new therapeutic strategy with rapid, sustained and safe antidepressant effects in late-life depression with inadequate antidepressant response.

**Funding:**

This study was supported by a grant from the French Ministry of Health and Prevention.

**Research in context**

**Evidence before this study**

We searched PubMed from database inception to December 31, 2025, for articles published in English, using the search terms “nitrous oxide”, “N_2_O” and “depression”. We also screened the reference lists of relevant articles to identify additional studies. Our search yielded 14 relevant results. Previous early-phase randomised controlled trials (RCTs) found that Nitrous Oxide (N_2_O) showed rapid and reproducible antidepressant effects in young adults with depression and an inadequate antidepressant response. These antidepressant effects generally lasted at least 24 h and, in some studies, persisted for up to one or two weeks after a single administration. N_2_O has several pharmacological properties that could make it a particularly promising candidate as a novel antidepressant therapy for older adults. These advantages include its rapid efficacy, its metabolism solely by the lungs, a robust safety profile established over decades, minimal drug interactions, few contraindications, and its ability to efficiently cross the blood–brain barrier. Despite these compelling advantages, no study to date has specifically evaluated its efficacy and tolerability as a fast-acting antidepressant in a population of older adults with depression and an inadequate antidepressant response.

**Added value of this study**

This multicentre, randomised, double-blind, placebo-controlled trial in late-life depression with an inadequate antidepressant response provides the first robust evidence that a single 50% N_2_O/50% O_2_ inhalation, compared with placebo (medical air), can produce a clinically meaningful, rapid reduction in depressive symptoms that is sustained over a two-week period. This trial demonstrated the feasibility of N_2_O inhalation for late-life depression with inadequate antidepressant response and confirmed its favourable benefit-to-harm ratio in this population.

**Implications of all the available evidence**

In late-life depression with inadequate antidepressant response, a condition where current options are limited by age-related physiological changes, a single 50%N_2_O/50%O_2_ inhalation offers a pragmatic, rapidly acting option that can deliver clinically meaningful symptom relief within days and persist for a minimum of two weeks. Adverse effects are typically mild and short-lived, although routine screening for emergent hypomania or psychotic symptoms remains prudent. Future studies may focus on determining the best strategies for induction and long-term maintenance of the antidepressant effect, investigating the efficacy of N_2_O in patients with non-resistant depression or more severely resistant depression, assessing the generalisability of these findings to other specific populations, such as patients with greater cognitive impairment or higher comorbidity burdens, conducting larger, multi-dose, and longer-follow-up trials to define durability, optimal scheduling, and comparative effectiveness.

## Introduction

There is limited evidence available regarding treatment strategies for late-life depression with inadequate antidepressant response, a condition that imposes a substantial personal and societal burden through persistent disability, loss of independence, increased healthcare utilisation, caregiver strain, and excess mortality.^1^, ^2^, ^3^ A recent systematic review, for instance, reported only weak support for ketamine and aripiprazole augmentation, and even weaker evidence for transcranial magnetic stimulation, P-glycoprotein modulation, and cognitive remediation.^4^ The requirement for novel therapeutic strategies in late-life depression is further underscored by the specific clinical and biological shifts associated with ageing. Indeed, age-related physiological changes (including slower metabolism,^5^ changes in pharmacokinetics and pharmacodynamics,^6^ increased drug sensitivity,^6^ and the high prevalence of polypharmacy^7^ and medical comorbidities^8^) substantially limit the efficacy of antidepressant strategies^9^ and increase the risk of severe adverse effects in this population.^10^ Given these age-related specificities, the limited number of strategies with proven efficacy and the high burden of late-life depression with inadequate antidepressant response, the development of new, evidence-based therapeutic interventions for this indication remains a clear priority.

Nitrous oxide (N_2_O) is a promising new antidepressant for older adults with depression, owing to a potentially favourable benefit-to-harm ratio. Evidence from studies in younger adults suggests that it has rapid and lasting antidepressant effects, with pathophysiological studies^11^ and reviews of randomised controlled trials (RCTs) finding benefits that lasted up to 2 weeks.^12^ Furthermore, a recent large RCT, despite being underpowered for its primary outcome, showed significant response and remission rates after four weekly sessions.^13^ Similar to ketamine, the antidepressant properties of N_2_O are thought to involve the glutamatergic system and specific downstream mechanisms.^14^ Specifically, N_2_O acts as a non-competitive NMDA receptor (NMDAR) antagonist, triggering plasticity pathways that sustain antidepressant effects beyond drug washout.^15^ Convergent evidence suggests the involvement of layer V prefrontal pyramidal neurons, providing a plausible microcircuit substrate that links NMDAR-dependent effects to broader network reconfiguration.^16^ At the network level, the antidepressant effects of N_2_O were associated with a reduction in subgenual Anterior Cingulate Cortex-praecuneus functional connectivity, within the default mode network.^17^

N_2_O is particularly well tolerated for older adults due to its unique pharmacological properties. It is eliminated primarily through the lungs, bypassing age-related declines in liver metabolism and kidney excretion.^18^^,^^19^ Its extensive, decades-long use in anaesthesiology and pain management has established a strong safety profile^20^ and it is generally well tolerated, even in frail older adults.^21^ N_2_O also has minimal drug interactions, few contraindications (mostly related to pulmonary disease), and efficiently crosses the blood–brain barrier.^19^^,^^20^^,^^22^ Also, because N_2_O inactivates vitamin B_12_, caution is warranted in susceptible older adults, as repeated exposure may lead to neuropathy, myelopathy, or gait disturbances.^23^ Despite these compelling advantages, no study to date has specifically evaluated its efficacy and tolerability as a fast-acting antidepressant in this population.

The aim of our study was to evaluate the efficacy and safety of a single 1-h exposure to a 50% N_2_O/50% O_2_ mixture in late-life depression with inadequate antidepressant response. The study was conducted as a randomised, multicentre, controlled trial with a 2-week follow-up period.

## Methods

### Study design

This study was a parallel-arm, multicentre, superiority, randomised, placebo-controlled trial, as an add-on to usual psychotropic treatments, conduced in the old-age psychiatry departments of 3 French University Hospitals. Participants were randomly assigned in a 1:1 ratio to receive either the active treatment (a mixture of 50% N_2_O and 50% O_2_) or the placebo (medical air, 78% N_2_/21% O_2_) during a 1-h inhalation session and were then followed for 2 weeks.

The study received approval from the Ethics Committee (CPP du Sud-Ouest et Outre-Mer-N°21.02.02.710000) and the French Agency ANSM (n°2019-004984-31), and all participants provided written informed consent. The study protocol and the trial was officially registered on ClinicalTrials.gov (NCT05007028).

### Participants

To be eligible, participants had to meet the following criteria: 1) Age between 60 and 90 years, 2) Diagnosis of a major depressive episode (MDE) according to DSM-5, 3) Montgomery-Åsberg Depression Rating Scale (MADRS) score greater than 20, 4) Resistance to at least one adequately conducted antidepressant treatment for at least 8 weeks for the current MDE episode, as evaluated by the Massachusetts General Hospital Antidepressant Treatment Response Questionnaire (MGH-ATRQ), 5) Able of receiving the gas mixture via facial mask, 6) Provide written informed consent, 7) Affiliated with the French national health insurance system.

Participants were excluded if they met any of the following criteria: 1) Diagnosis of bipolar disorder, schizophrenia, major neurocognitive disorder, or substance use disorder, 2) A Mini-Mental State Examination (MMSE) score lower than 24, 3) Unstable medical conditions, particularly unstable neurological or cardiac disorders that could interfere with N_2_O diffusion, or any recent and unexplained neurological abnormality, 4) Presence of active and significant psychotic symptoms, as determined by the investigator, 5) N_2_O contraindications or any condition where trapped air could be hazardous, including: pneumothorax, emphysema, bowel obstruction, intracranial hypertension, known and untreated deficiency in vitamin B_12_ or B_9_, requirement for pure oxygen ventilation, altered consciousness preventing cooperation, head trauma, gas embolism, diving accident, abdominal gas distension, or recent (within 3 months) intraocular gas injection 6) Legal incapacity or any other circumstance preventing the participant from understanding the nature, purpose, or consequences of the study, 7) Already enrolled in another investigational drug study or within an exclusion period following participation in a previous clinical trial.

### Randomisation

Participants were randomised using *Ennov Clinical©*, an online central randomisation procedure. Randomisation occurred only after a participant was recruited into the trial. All eligibility criteria must have been collected and met. The allocation sequence was generated using *SAS©* by a statistician not involved in the recruitment or follow-up of the participants. Randomisation was stratified by the centre.

Participants, investigators, and outcome assessors were blinded to the allocated group. The nurse administering the gas and the anaesthetist monitoring the participant were aware of group assignments. Blinding effectiveness was assessed at the end of the trial by asking participants whether they believed they knew their assigned treatment group, and specifically whether they thought they had received nitrous oxide or medical air.

### Study procedures

Each participant attended five visits and was asked to continue usual depression treatment with no change in psychotropic medication dose during the study. Before formal enrolment, individuals were given a minimum of a 48-h reflection period after receiving detailed study information. The baseline visit marked the official enrolment; participants provided written informed consent. During this comprehensive visit, a clinical and psychometric evaluation was conducted, including the collection of demographic data and documentation of medical and surgical history using the Cumulative Illness Rating Scale (CIRS). The level of treatment resistance for the depressive episode was assessed using the Maudsley Staging Method (MSM). A comprehensive battery of psychometric scales was administered, including MADRS, MGH-ATRQ to screen for antidepressant use in the current MDE, MMSE to evaluate cognitive function. Additionally, a thorough neurological, cardiovascular, and pulmonary examination was performed, and vital signs were measured to rule out any somatic conditions. Following the evaluations, participants were randomised to either the N_2_O or placebo group, and the exposure to the assigned intervention was administered within 7 days after the baseline assessment (typically within 1–2 days).

### Gas exposure

Gas exposure was conducted using cylinders containing either a 50% N_2_O/50% O_2_ mixture for the N_2_O group, or medical air (78% N_2_/21% O_2_) for the placebo group. To ensure participant blinding, the study nurse carefully concealed the gas cylinder behind a screen before participants entered the room. All participants underwent a 1-h inhalation session. Nitrous oxide was administered using a standard anaesthetic face mask equipped with an adjustable cushioned seal to minimise leakage and ensure an adequate fit. The mask was held in place by trained clinical staff throughout administration, allowing for continuous monitoring of positioning and patient comfort, in accordance with the standard of care for medical N_2_O use. In addition, nitrous oxide was delivered using a calibrated gas delivery system with controlled flow rates, and administration was conducted in a clinical setting with appropriate room ventilation, consistent with standard anaesthetic safety practices. While minimal entrainment of room air cannot be entirely excluded, the use of a well-fitted mask and continuous supervision were intended to limit dilution of the inspired gas concentration. The gas flow was generally maintained between 6 and 9 L/min, with the nurse adjusting this parameter during the procedure based on the participant's respiratory rate. Vital signs (oximetry, blood pressure, heart rate, and respiratory rate) were continuously monitored, along with behavioural and neurological signs. After exposure, participants received 15 min of 100% oxygen to reduce the risk of hypoxia and were monitored for at least 2 h. A final medical check was conducted by the supervising physician before discharge.

The choice of medical air as the placebo control was guided by several considerations. Medical air is straightforward to implement, preserves participant comfort and safety, and closely resembles the physical conditioning of the preconditioned 50% N_2_O/50% O_2_ mixture bottles. An alternative approach would have been the use of a 50% O_2_/medical air mixture; however, although short-term administration of 50% oxygen is generally well tolerated in older adults, it may pose risks (particularly oxygen-induced hypercapnia) in geriatric populations with underlying pulmonary insufficiency.^16^

### Outcome measures

We collected data at baseline, 2 h, 24 h, 1 week, and 2 weeks after gas exposure for each participant. Primary outcomes assessed depressive symptoms changes based on MADRS scores at 2 weeks, with lower scores indicating fewer symptoms. We chose the difference in MADRS at week 2 as the primary outcome because, while previous studies reported the largest effects of N_2_O at 2 and 24 h post-treatment, assessing a longer-term effect is more clinically relevant and better accounts for the natural fluctuations of depression. Other analyses than MADRS at 2 weeks, including MADRS changes at 2 h, 24 h, and 1 week, were considered exploratory, and no adjustment for multiple comparisons was applied.

Secondary outcomes for treatment efficacy included: 1) Hamilton Depression Rating Scale (HDRS), to measure depression severity, 2) Clinical Global Impression Severity (CGI-S), to assess the severity of the patient's illness, 3) Clinical Global Impression Improvement (CGI-I), to assess illness improvement, 4) Quick Inventory of Depressive Symptomatology-Self Report (QIDS-SR), a self-assessment of depression severity, 5) State-Trait Anxiety Inventory, Form Y-A (STAI-Y-A), a self-assessment of anxiety symptoms, 6) Visual Analogue Scale (VAS), a self-rating of well-being ranged from 0 (“worst possible wellbeing”) to 100 (“best possible wellbeing”).^24^

Safety outcomes were assessed using: 1) Suicide Ideation Scale (SSI), to measure the severity of suicidal ideation 2) Young Mania Rating Scale (YMRS) to assess the severity of manic symptoms, 3) Clinician-Administered Dissociative States Scale (CADSS), to assess the presence of dissociative symptoms, 4) 4 items of the Brief Psychiatric Rating Scale (BPRS) to evaluate the severity of psychotic symptoms. Adverse events were continuously monitored and documented during and immediately after gas exposure.

### Statistical analysis

Based on the results of the pilot trial from Nagele et al., we expect a between group difference of 2.7 points in the MADRS (standard deviation SD = 5) 2 weeks after N_2_O exposure. The choice of a 2.7-point difference on the MADRS was intentionally conservative and was informed by the proof-of-concept randomized controlled trial of N_2_O in TRD by Nagele et al. (2015).^11^ In that crossover study, a single 1-h administration of 50% N_2_O/50% O_2_ produced a between-condition difference of approximately 5.5 points on the HDRS-21 at 24 h compared with placebo. Given key differences between that study and the present trial (including a parallel-group design, a bipolar depression population, and the use of MADRS rather than HDRS-21) we conservatively selected a target effect corresponding to roughly half of the previously observed between-group difference, resulting in a 2.7-point MADRS difference. This estimate reflects a clinically meaningful yet cautious assumption and aligns with commonly accepted minimal clinically important differences of 2–3 MADRS points in antidepressant trials.^25^ Based on this between-group difference and using the approach formalised by Vickers (Vickers, 2003) and by Borm (Borm et al., 2007) for the calculate of the sample size for a trial with repeated measures, 28 participants per group was required to achieve 90% power with a 2-sided α of 0.05, accounting for 5 measures (baseline and 4 follow-ups) and assuming a within subject correlation of 0.5.^26^^,^^27^ The recruitment target was increased to 30 participants per group.

The statistical analyses were performed according to a statistical analysis plan.

Baseline characteristics were reported with mean and SD or with median and interquartile range for quantitative variables and with number and percentage for qualitative variables. No statistical comparison was performed on baseline characteristics.

The primary outcome was analysed according to the modified intention-to-treat principle. All the randomised participants were included with respect to their allocated group except those who withdrew consent and who refuse the use of their data. The analysis of the MADRS was based on a mixed model for repeated measures, using restricted estimated maximum likelihood method. We include in the model fixed effects for the time of measurement (treated as categorical), for the treatment group (N_2_O vs placebo), for the interactions between time and group and a random effect for the subject. This subject random effect allows modelling the correlation between repeated measurements from a same participant upon the assumption of a compound symmetry variance-covariance matrix. The difference between groups in change from baseline at each time point was modelled with the interaction terms between the groups and the times of measurement. Estimates were reported with a two-sided 95% confidence interval, with the associated p-value and with Cohen's D for effect size. A particular attention was given to the interaction term of the 2 weeks measurement of the MADRS, considered as our primary endpoint. The other measurements of the MADRS (at 2 h, 24 h and 1 week) were considered as secondary outcomes as no adjustment for multiple comparisons was planned. Robustness of departure from the compound symmetry assumption was tested in a sensitivity analysis, using a model with unstructured variance-covariance matrix. For this sensitivity analysis we used a constrained longitudinal model to investigate whether or not of our estimates were sensitive to an adjustment on the MADRS score at baseline (estimates of fixed effects for this sensitivity analysis and associated p-value are reported in the supplementary Appendix p 2). In post hoc analysis, the proportions of responders (the MADRS score at baseline divided by at least 2 at 2 weeks) and of remitters (the MADRS score at 2 weeks equal or lower than 10) were compared between-groups using $\chi^{2}$ test or Fisher's exact test, depending on how the validity conditions were met are not.

The others secondary outcomes were analysed using similar model than for the primary analysis of the MADRS except for the CGI-I scale where the scores were dichotomised in very much better and much better vs minimally better to very much worse. Comparisons between groups at each time of planned measurement were then performed using $\chi^{2}$ test or Fisher's exact test. The analyses of all those others secondary outcomes were also considered as exploratory, and no adjustments were made for multiple comparisons.

The safety outcomes (YMRS, CADSS and BPRS) were analysed using models similar to those for the MADRS. Adverse and serious adverse events were reported with number and percentage.

A *p*-value below 0.05 was considered significant. Statistical analyses were performed using R software version 4.1.2 (R Core Team, 2021, Austria).

### Role of the funding source

The funder of the study had no role in study design, data collection, data analysis, data interpretation, or writing of the report.

## Results

### Recruitment, randomisation, participant flow and baseline characteristics

**Fig. 1:**
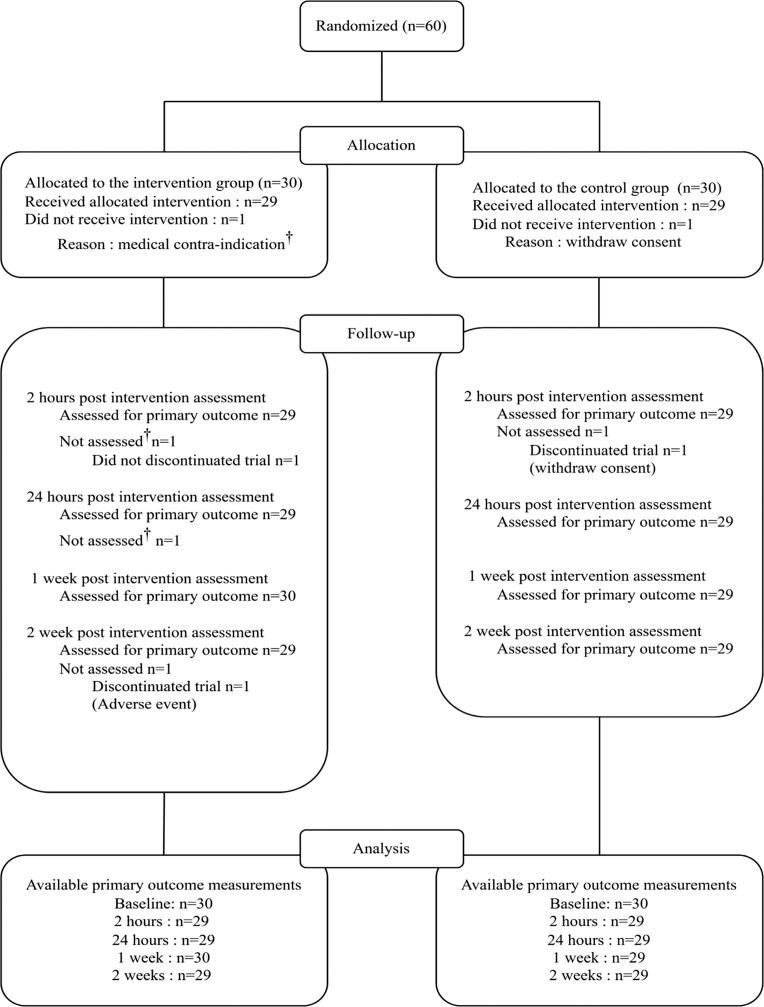
Flowchart. Gas exposure was contra-indicated due to the occurrence of an acute event (panic attack) just before starting gas exposure. The participant was not assessed at 2 h and at 24 h but was assessed at 1 week and at 2 weeks.

A total of 60 participants were randomised to receive either nitrous oxide (N_2_O, n = 30) or medical air (placebo, n = 30), as detailed in the CONSORT flow diagram (Fig. 1). Inclusion was carried out between November 17, 2021, and December 3, 2024. One participant in the placebo group withdrew consent immediately after randomisation, and no post-baseline data were collected for this individual. In the N_2_O group, one participant did not receive the treatment due to a newly identified medical contraindication just before gas administration. This participant was assessed at baseline, 1 week and 2 weeks follow-up visits. Another participant in the N_2_O group discontinued participation before the 2 weeks assessment due to a worsening of underlying medical condition, deemed unrelated to study treatment. It leads to an attrition rate of 3.3% for the primary endpoint. Baseline characteristics were balanced between groups, with no clinically meaningful differences (Table 1).

**Table 1:** Baseline characteristics in the intervention group (N_2_O–Nitrous Oxide) and in the control group.

Characteristic	N_2_O	Control
	n = 30	n = 30
Age (years)	74.5 (7.4)	70.2 (8.1)
Female sex	20 (66.7%)	21 (70%)
BMI (kg/m^2^)	25.6 (4)	28.5 (7.3)
SBP (mmHg), n = 59 (30/29)^a^	135.2 (18.1)	134.7 (19.6)
DBP (mmHg), n = 59 (30/29)^a^	74.9 (13.2)	77.6 (12.3)
CIRS	8.7 (4.5)	7.6 (4.9)
Early-Onset Depression	21 (70%)	21 (70%)
Number of previous MDE, n = 59 (29/30)^a^	3 [2; 5]	3 [2; 5]
Duration of MDE (weeks)	99.7 (84.8)	116.1 (95.5)
Maudsley Staging Method	8.8 (2.6)	8.4 (2.1)
MMSE	27 (2.5)	27.5 (1.9)
Current medications, n = 59 (29/30)^a^		
Antidepressants		
SSRI	11 (37.9%)	10 (33.3%)
SNRI	14 (48.3%)	11 (36.7%)
Tricyclic	1 (3.4%)	3 (10%)
Tetracyclic	8 (27.6%)	10 (33.3%)
Vortioxetine	2 (6.9%)	3 (10%)
Others		
Antiepileptic	4 (13.8%)	9 (30%)
Benzodiazepines	16 (55.2%)	15 (50%)
MADRS	31.6 (5.9)	30.0 (6.9)
HDRS	21.4 (5.7)	20.1 (6.8)
CGI severity	5.3 (0.8)	5.1 (0.9)
QIDS-SR	20.4 (6.5)	19.0 (6.1)
STAI-Y-A	57.9 (11.9)	53.0 (14.1)
VAS	31.4 (15.7)	32.6 (19.6)

For quantitative variables, values are mean and standard deviation or median and interquartile range. For categorical variables, values are number and percentage.

BMI: Body Mass Index; CIRS: Cumulative Illness Rating Scale; CGI: Clinical Global Impression; DBP: Diastolic Blood Pressure; SBP: Systolic Blood Pressure; HDRS: Hamilton Depressive Rating Scale; MADRS: Montgomery *Å*sberg Depression Rating Scale; MDE: Major Depressive Episode; QIDS-SR: Quick Inventory of Depressive Symptomatology Self-Report; SNRI: Serotonin-Norepinephrine Reuptake Inhibitor; SSRI: Selective Serotonin Reuptake Inhibitor; STAI-Y-A: State-Trait Anxiety Inventory; VAS: Visual Analogue Scale ranged from 0 (“worst possible wellbeing”) to 100 (“best possible wellbeing”).

a Numbers per group are indicated when a measurement was missing for at least one participant.

### Efficacy outcomes

**Fig. 2:**
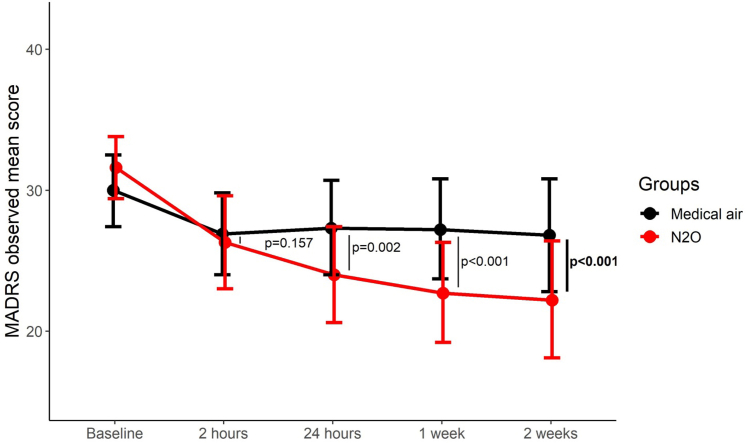
Longitudinal plot of means of MADRS scores by group. Medical air corresponds to the control group. Points are observed mean scores of the MADRS (and not estimated from the model) at each time of planned measurement. Error bars are 95% confidence intervals. The indicated p-values are for the between groups differences at each time point estimated with a mixed model for repeated measures. In bold is the primary endpoint (at 2 weeks), others time points (2 h, 24 h and 1 week) were secondary endpoints. MADRS, Montgomery Asberg Depression Rating Scale.

For the primary endpoint, the between groups difference in MADRS change from baseline at 2 weeks was −6.2 points (95% CI, −9.1 to −3·4; p < 0.001; Cohen's D = −0.71). For the others measures of the MADRS (secondary endpoints), the between-group difference in change from baseline also favoured N_2_O at 24 h (−4.8 points; 95% CI, −7.6 to −1.9; p = 0.002, Cohen's D = −0.54) and at 1 week (−6.0; 95% CI, −8·8 to −3·1; p < 0·001; Cohen's D = −0.68) with no significant difference at 2 h post-inhalation (Fig. 2; results from linear mixed models provided in Table 2). At 2 weeks, response occurred in 8 of 29 participants (27.6%) in the N_2_O group vs 4 of 29 (13.8%) with placebo, without significant differences, p = 0.195; and remission occurred in 4 of 29 (13.8%) vs 3 of 29 (10.3%), respectively, without significant differences, p > 0.999.

**Table 2:** Results of the analysis of the MADRS.

Effect	Estimate	95% CI	p-value
Intercept	30.0	[26.8; 33.1]	<0.001
Time effects			
2 h	−3.3	[−5.3; −1.2]	0.002
24 h	−2.8	[−4.9; −0.8]	0.007
1 week	−2.9	[−5; −0.9]	0.005
2 weeks	−3.4	[−5.4; −1.3]	0.002
Group effect, N_2_O	1.7	[−2.8; 6.1]	0.466
Time × group interactions			
2 h × N_2_O	−2.1	[−5; 0.8]	0.157
24 h × N_2_O	−4.8	[−7.6; −1.9]	0.002
1 week × N_2_O	−6.0	[−8.8; −3.1]	<0.001
2 weeks × N_2_O^a^	−6.2	[−9.1; −3.4]	<0.001

Values are estimates of the fixed effects from a mixed model for repeated measures. Time effects model the effect, for each time point of the follow up, of medical air exposure on the depressive symptoms measured with the MADRS scale. Time × group interactions model the additional effect of the N_2_O exposure on depressive symptoms (the between group differences at each time point).

a Time × group interaction effect at week 2 was the primary endpoint, others time points were secondary endpoints.

**Fig. 3:**
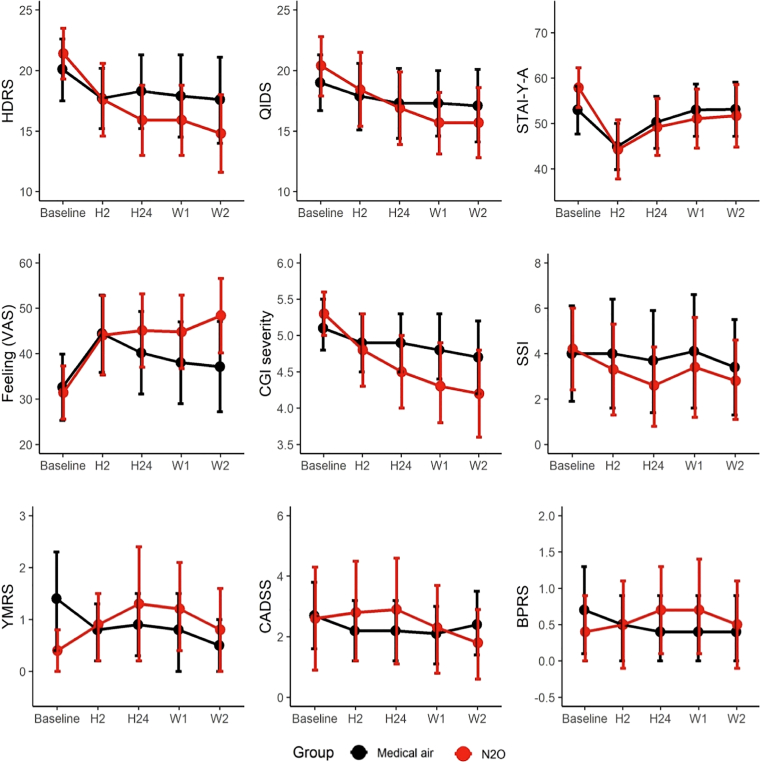
Longitudinal plot of means of the secondary outcomes scores by group. Points are observed mean scores (and not estimated from the linear mixed effect model) at each time of planned measurement. Error bars are 95% confident intervals. From top left to bottom right: HDRS, QIDS, STAI-Y-A, VAS, CGI-S, CGI-I, SSI, YMRS, CADSS, BPRS. HDRS, Hamilton Depressive Rating Scale; QIDS-SR, Quick Inventory of Depressive Symptomatology Self-Report; STAI-Y-A, State-Trait Anxiety Inventory; VAS, Visual Analogue Scale; CGI-S, Clinical Global Impression—Severity; CGI-I, Clinical Global Impression—Improvement; SSI, Suicide Ideation Scale; YMRS, Young Mania Rating Scale; CADSS, Clinician-Administered Dissociative States Scale; BPRS, Brief Psychiatric Rating Scale. H2 is for the assessment at 2 h post exposure, H24 for the assessment at 24 h post exposure, W1 for 1 week and W2 for 2 weeks post exposure.

Regarding others secondary outcomes (Fig. 3; Appendix pp 3 and 4), between-group differences in change from baseline at 2 weeks favoured N_2_O for HDRS (−4.2; 95% CI, −6.6 to −1.9; p < 0.001; Cohen's D = −0.55), QIDS (−2.8; 95% CI, −5.5 to −0.2; p = 0.042; Cohen's D = −0.39), STAI-Y-A (−6.7; 95% CI, −13.1 to −0.3; p = 0.046; Cohen's D = −0.43), and CGI-S (−0.5; 95% CI, −0.9 to −0.1; p = 0.01; Cohen's D = −0.45); VAS improvement was greater at week 2 in the N_2_O group (11.5; 95% CI, 3.5–19.5; p = 0.006; Cohen's D = 0.52). The proportion with marked improvement on CGI-I was higher with N_2_O at 1 week only (N_2_O group: n = 9, 30.0%; control group: n = 2, 6·9%, Fisher's exact test, p = 0.042) (Appendix p 7).

### Safety outcomes

During follow-up, one serious adverse event occurred, unrelated to study treatment. Non-serious events (anxiety, dizziness, numbness) occurred during inhalation were more common with N_2_O, but all resolved on the same day (Table 3).

**Table 3:** Duration and estimated volume of gas exposure, adverse event during gas exposure, vitamin B12 monitoring and blinding check (participants were asked if they guess the treatment they received).

	N_2_O	Control
	n = 29	n = 29
Duration of exposure, min	60.1 (3)	60 (0)
Volume administered, litres	543.8 (47.7)	513.7 (106.6)
Adverse event during exposure		
Anxiety	5 (17.2%)	3 (10.3%)
Numbness	4 (13.8%)	0 (0%)
Dizziness	3 (10.3%)	0 (0%)
Panic attack	2 (6.9%)	0 (0%)
Nausea	1 (3.4%)	1 (3.4%)
Headache	0 (0%)	2 (6.9%)
Others^a^	2 (6.9%)	1 (3.4%)
Adverse event after exposure		
Panic attack	3 (10.3%)	1 (3.4%)
Headache	1 (3.4%)	0 (0%)
Dizziness	0 (0%)	1 (3.4%)
Weakness	1 (3.4%)	0 (0%)
Lightheadedness	1 (3.4%)	0 (0%)
Tinnitus	1 (3.4%)	0 (0%)
Falling	0 (0%)	1 (3.4%)
Difficulty urinating	1 (3.4%)	0 (0%)
Abdominal pain	0 (0%)	1 (3.4%)
Nausea	1 (3.4%)	0 (0%)
Diarrhoea	0 (0%)	1 (3.4%)
Constipation	1 (3.4%)	0 (0%)
Vitamin B12^c^, pmol/L		
Baseline (n = 29, n = 30)	368.1 (141.7)	347.3 (154.7)
24 h post treatment (n = 27, n = 27)	379.0 (167.5)	349.0 (151.1)
Blinding check (n = 24/n = 27)		
Thinks they know which the treatment they received^b^		
No	7 (29.2%)	8 (29.6%)
Yes	17 (70.8%)	19 (70.4%)
If yes		
Answers N_2_O	11 (64.7%)	2 (10.5%)
Answers medical air	6 (35.3%)	17 (89.5%)

a Participants were asked at the end of the 2-week visit.

b Value are mean and standard deviation.

Dissociative symptoms (CADSS) and suicidality (SSI) did not change significantly in either group, whereas manic symptoms (YMRS) were significantly higher in the N_2_O group at 24 h, 1 week and 2 weeks. Psychotic symptoms (BPRS) were also significantly higher in the N_2_O group at 24 h and week 1 (Appendix pp 5 and 6).

### Blinding effectiveness

Correct treatment guesses were reported by 11 of 24 participants (46%) in the N_2_O group and 17 of 27 (63%) in the placebo group (Table 3).

## Discussion

We found for the first time that a single 1-h administration of N_2_O was safe and provided rapid and sustained antidepressant effects in late-life depression with inadequate response to standard treatments. These findings extend the positive outcomes previously observed in younger populations^11^^,^^28^ to an older population. Specifically, a recent meta-analysis of seven trials in young adults reported rapid antidepressant effects that typically emerged at 2 h, peaked within 24 h, but were no longer significant by one week.^12^ In contrast, our results in late-life depression show a clinically meaningful response lasting up to two weeks, suggesting a more durable effect in this population.

Notably, the antidepressant effect was not significantly apparent at 2 h post-administration, differing from the immediate response often observed in younger adults. In our cohort, significant differences emerged at 24 h and persisted through two weeks. This delay may be due to the minimal dissociation experienced by our participants. Indeed, evidence from ketamine studies suggests that acute dissociative effects contribute to the initial response at 2 h.^29^ Alternatively, older adults may be less sensitive to N_2_O-induced neurobiological mechanisms, where NMDA receptor antagonism requires more time to trigger the downstream synaptic plasticity and consolidation processes necessary for clinical improvement.^30^

Regarding safety, the treatment was well tolerated, with a profile comparing favourably with younger cohorts. While common adverse effects in young adults typically include nausea, dizziness, and transient dissociation,^12^ our participants experienced only minimal symptoms. However, we observed potential risks of anxiety, manic or delusional symptoms that have not been previously reported in N_2_O trials of younger adults, even among those with bipolar disorder.^31^ Specifically, we observed mean increases of 1.3 on the YMRS (scale 0–60) and 0.5 on the 4-item BPRS (scale 0–16) from near-zero baselines. Given these low scores and the absence of behavioural disturbances, these changes are unlikely to be clinically meaningful. Nevertheless, our findings suggest that manic and psychotic symptoms warrant careful monitoring in older populations.

Overall, the safety of N_2_O in our cohort was consistent with its established profile in older and frail patients across other clinical contexts.^20^ Notably, we observed no clinically meaningful reductions in vitamin B12 levels. This is significant, as older adults are often predisposed to B12 deficiency and its associated neurological or haematological complications. However, as deficiency is more likely to arise from repeated exposure, future studies involving multiple administrations should monitor B12 levels closely. Measuring homocysteine may also be advisable, as it provides a more sensitive marker for acute N_2_O-induced metabolic changes.^13^ Finally, the potential long-term impact of N_2_O on cognition remains unknown and was not assessed in our study. The long-term safety profile in late-life depression therefore remains insufficiently characterised, underscoring the need for further research and careful clinical monitoring.

Interestingly, the overall high tolerability of N_2_O may have enhanced blinding integrity, with fewer than half of participants correctly guessing their treatment allocation. By contrast, studies in younger adults have reported that over three-quarters of participants correctly identified their treatment arm.^32^ A major challenge to blinding in psychedelic studies, including those involving (es)ketamine, is the presence of acute dissociative effects, which account for a substantial proportion of blinding failures.^33^ Therefore, the low incidence of dissociative symptoms observed in our study, which aligns with evidence that older adults are generally less susceptible to the dissociative effects of N_2_O,^34^ likely contributed to the effective blinding.

Within the current landscape of treatments for late-life depression with inadequate antidepressant response, N_2_O offers a distinct clinical option. Compared with conventional antidepressants or augmentation strategies, such as aripiprazole, N_2_O provides a more rapid onset of action and superior tolerability. While aripiprazole may expose older adults to serious risks, including parkinsonism, sedation, falls, and cerebrovascular events, N_2_O's primary risks differ in nature and severity. Furthermore, N_2_O may offer a more favourable safety profile than (es)ketamine, with a lower incidence of clinically significant dissociation and hypertension. Although both treatments require nurse supervision and the ideal frequency for repeated N_2_O remains unestablished, N_2_O may offer a more manageable monitoring burden. Interestingly, our participants may have exhibited a lower level of treatment resistance at baseline than those in typical (es)ketamine trials. This further suggests that N_2_O could be a suitable intervention not only for those with established treatment-resistant depression, but also for patients in the early-to-medium stages of resistance.

This trial has several limitations. First, the study investigated only a single administration with follow-up limited to two weeks; consequently, the effects of repeated dosing and longer-term outcomes remain uncharacterised. Given the modest rates of response and remission, some patients may require multiple administrations to achieve full benefit. In addition, apart from the MADRS difference at Week 2, all other analyses were considered exploratory and should be interpreted with caution. Second, we used medical air as the placebo rather than a mixture of 50% O_2_/medical air. Accordingly, our findings should be interpreted as reflecting the effects of the 50% N_2_O/50% O_2_ mixture rather than N_2_O alone. We cannot exclude a potential contribution of elevated oxygen to the observed antidepressant effects, as suggested by prior studies of oxygen exposure, although oxygen-related mood benefits are more commonly reported with repeated administrations.^35^ Future studies may consider using an oxygen concentration-matched control, such as 50% O_2_/medical air. Third, our inclusion definition of depression with inadequate response required only one prior antidepressant failure, and participants had on average moderate resistance. The efficacy of N_2_O in patients with more severe or chronic resistance is uncertain. Fourth, although the protocol required stable psychotropic treatment, a few clinically driven changes occurred: lithium was added in two control-group participants, and lorazepam in two participants (one per group, with the control-group case overlapping a lithium initiation). Finally, participants had relatively low baseline cognitive impairment and a modest comorbidity burden, which may limit generalisability to frailer, more medically complex older adults, and the absence of formal post-exposure cognitive monitoring represents an additional limitation that should be addressed in future studies.

In conclusion, we demonstrated that a 1-h administration of 50% N_2_O/50% O_2_ has rapid and durable antidepressant effects in late-life depression with inadequate antidepressant response, with a good tolerability profile. While future studies are needed to determine the best strategies for induction and long-term maintenance, our findings represent a significant step forward in treating a serious disorder that urgently requires effective and safe therapies, especially in a population where current options are limited by age-related physiological changes.

## Contributors

TD conceived, designed and coordinated the study. AS and TD drafted the manuscript. PP and ALG accessed and verified the data and performed the statistical analyses. TD, AS, SB, GR, AV, QG, JAN, VL, PB, WEH and VC contributed to patient recruitment and data acquisition. VG and WEH led the clinical research centre. MSA, HB, NA, and FE provided pharmacological expertise.

All authors critically revised the manuscript for important intellectual content and approved the final version for submission.

## Data sharing statement

Anonymised individual participant data will be made available to researchers who provide a methodologically sound proposal for use in achieving the goals of the approved proposal. A prior written agreement must be signed with the University Hospital of Tours and the coordinator of the study.

## Declaration of interests

TD reports holding shares in Synaptys Neuroscience, has served as a consultant for Bristol Myers Squibb and has received personal fees from Janssen and Lundbeck, unrelated to the submitted work.

WEH reports personal fees from Air Liquide, Boehringer-Ingelheim, Chugai, EISAI, Idorsia, Jazz Pharmaceuticals, Johnson & Johnson, Lundbeck, Mindforce Game Lab, Novartis, Otsuka, UCB, unrelated to the submitted work.

All other authors declare no competing interests.

## Appendix A Supplementary data

Appendix

Clinical Trial Protocol

Statistical Analysis Plan
