# Supplementary material for: Nitrous oxide for late-life depression with inadequate antidepressant response: a randomised controlled trial
Source: eClinicalMedicine. 2026 Apr 2;94:103860. doi: 10.1016/j.eclinm.2026.103860 (PMC13084366; doi:10.1016/j.eclinm.2026.103860)
Supplement: Appendix [file mmc1.docx]

**Supplementary appendix**

[Table S1. Results from a sensitivity analysis concerning the MADRS using a constrained longitudinal model with a unstructured variance covariance matrix. 2](#_Toc220011632)

[Table S2. Results of the analysis of the secondary outcomes 3](#_Toc220011633)

[Table S3. Results of the analysis of the scales used for the assessment of psychiatric adverse events. 5](#_Toc220011634)

[Table S4. Results of the Clinical Global Impression Improvement scale scores. 7](#_Toc220011635)

| **Table S1.** Results from a sensitivity analysis concerning the MADRS using a constrained longitudinal model with a unstructured variance covariance matrix. | | | |
| --- | --- | --- | --- |
| **Effect** | **Estimate** | **95% CI** | **p-value** |
| *Intercept* | 30.8 | [29.2; 32.4] | **<0.001** |
| *Time effect* |  |  |  |
| *2 hours* | -3.3 | [-5; -1.6] | **<0.001** |
| *24 hours* | -2.9 | [-5.2; -0.6] | **0.014** |
| *1 week* | -3 | [-5; -0.5] | **0.02** |
| *2 weeks* | -3.3 | [-6.2; -0.5] | **0.021** |
| *Time × group interactions* |  |  |  |
| *2 hours × N_2_O* | -2.1 | [-4.5; 0.3] | 0.088 |
| *24 hours × N_2_O* | -4.5 | [-7.8; -1.3] | **0.007** |
| *1 week × N_2_O* | -5.8 | [-9.2; -2.3] | **0.001** |
| *2 weeks × N_2_O* | -6.2 | [-10.1; -2.3] | **0.002** |
|  |  |  |  |
|  | | | |

| **Table S2.** Results of the analysis of the secondary outcomes | | | |
| --- | --- | --- | --- |
| **Effect** | **Estimate** | **95% CI** | **p-value** |
| *Depressive symptoms (Hamilton depressive rating scale)* | | | |
| *Intercept* | 20.1 | [17.3; 22.8] | <0.001 |
| *Time effect* |  |  |  |
| *2 hours* | -2.3 | [-4; -0.6] | **0.008** |
| *24 hours* | -1.8 | [-3.4; -0.1] | **0.042** |
| *1 week* | -2.1 | [-3.8; -0.5] | **0.014** |
| *2 weeks* | -2.5 | [-4.2; -0.8] | **0.004** |
| *Group effect, N_2_O* | 1.3 | [-2.5; 5.2] | 0.504 |
| *Time × group interactions* |  |  |  |
| *2 hours × N_2_O* | -1.4 | [-3.8; 1] | 0.252 |
| *24 hours × N_2_O* | -3.6 | [-6; -1.3] | **0.003** |
| *1 week × N_2_O* | -3.4 | [-5.7; -1] | **0.006** |
| *2 weeks × N_2_O* | -4.2 | [-6.6; -1.9] | **<0.001** |
| *Depressive symptoms (Quick Inventory Depressive Symptoms)* | | | |
| *Intercept* | 19 | [16.4; 21.6] | <0.001 |
| *Time effect* |  |  |  |
| *2 hours* | -1.2 | [-3.1; 0.6] | 0.205 |
| *24 hours* | -1.8 | [-3.7; 0.1] | 0.067 |
| *1 week* | -1.4 | [-3.3; 0.5] | 0.16 |
| *2 weeks* | -2 | [-3.9; -0.1] | **0.042** |
| *Group effect, N_2_O* | 1.4 | [-2.3; 5] | 0.467 |
| *Time × group interactions* |  |  |  |
| *2 hours × N_2_O* | -0.8 | [-3.5; 1.9] | 0.556 |
| *24 hours × N_2_O* | -1.8 | [-4.5; 0.9] | 0.19 |
| *1 week × N_2_O* | -3.3 | [-6; -0.6] | **0.017** |
| *2 weeks × N_2_O* | -2.8 | [-5.5; -0.2] | **0.042** |
| *Anxiety (State Trait Anxiety Inventory)* | | | |
| *Intercept* | 53 | [47.5; 58.4] | <0.001 |
| *Time effect* |  |  |  |
| *2 hours* | -8 | [-12.5; -3.5] | **0.001** |
| *24 hours* | -2.6 | [-7.1; 1.9] | 0.264 |
| *1 week* | 0.2 | [-4.4; 4.8] | 0.939 |
| *2 weeks* | 0.5 | [-4.1; 5] | 0.847 |
| *Group effect, N_2_O* | 4.9 | [-2.8; 12.6] | 0.216 |
| *Time × group interactions* |  |  |  |
| *2 hours × N_2_O* | -5.3 | [-11.7; 1] | 0.107 |
| *24 hours × N_2_O* | -5.8 | [-12.2; 0.6] | 0.079 |
| *1 week × N_2_O* | -7.5 | [-13.9; -1.1] | **0.025** |
| 2 weeks × *N_2_O* | -6.7 | [-13.1; -0.3] | **0.046** |
| *Feeling (Visual Analogue Scale)* | | | |
| *Intercept* | 32.6 | [24.8; 40.4] | <0.001 |
| *Time effect* |  |  |  |
| *2 hours* | 12.3 | [6.6; 18] | **<0.001** |
| *24 hours* | 8 | [2.3; 13.7] | **0.007** |
| *1 week* | 5.9 | [0.2; 11.6] | **0.046** |
| *2 weeks* | 5 | [-0.7; 10.7] | **0.09** |
| *Group effect, N_2_O* | -1.1 | [-12.2; 9.9] | 0.842 |
| *Time × group interactions* |  |  |  |
| *2 hours × N_2_O* | 0.3 | [-7.7; 8.4] | 0.94 |
| *24 hours × N_2_O* | 5.6 | [-2.5; 13.6] | 0.18 |
| *1 week × N_2_O* | 7.4 | [-0.6; 15.4] | 0.074 |
| *2 weeks × N_2_O* | 11.5 | [3.5; 19.5] | **0.006** |
| *Clinical Impression (Clinical Global Impression scale - severity)* | | | |
| *Intercept* | 5.1 | [4.7; 5.6] | <0.001 |
| *Time effect* |  |  |  |
| *2 hours* | -0.3 | [-0.6; 0] | 0.064 |
| *24 hours* | -0.2 | [-0.5; 0] | 0.105 |
| *1 week* | -0.3 | [-0.6; 0] | **0.037** |
| *2 weeks* | -0.4 | [-0.7; -0.2] | **0.003** |
| *Group effect, N_2_O* | 0.1 | [-0.5; 0.7] | 0.671 |
| *Time × group interactions* |  |  |  |
| *2 hours × N_2_O* | -0.2 | [-0.6; 0.2] | 0.345 |
| *24 hours × N_2_O* | -0.5 | [-0.9; -0.1] | **0.016** |
| *1 week × N_2_O* | -0.6 | [-1; -0.2] | **0.003** |
| *2 weeks × N_2_O* | -0.5 | [-0.9; -0.1] | **0.01** |

Values are estimates of the fixed effects from mixed models for repeated measures.

| **Table S3.** Results of the analysis of the scales used for the assessment of psychiatric adverse events. Values are estimates of the fixed effects from linear regression mixed effect models. | | | |
| --- | --- | --- | --- |
| **Effect** | **Estimate** | **95% CI** | **p-value** |
| *Risk of suicide (Scale for Suicidal Ideation)* | | | |
| *Intercept* | 4 | [2; 6] | <0.001 |
| *Time effect* |  |  |  |
| *2 hours* | 0 | [-1.2; 1.1] | 0.952 |
| *24 hours* | -0.3 | [-1.4; 0.8] | 0.591 |
| *1 week* | 0.1 | [-1; 1.2] | 0.858 |
| *2 weeks* | -0.6 | [-1.7; 0.5] | 0.284 |
| *Group effect, N_2_O* | 0.2 | [-2.6; 3] | 0.889 |
| *Time × group interactions* |  |  |  |
| *2 hours × N_2_O* | -0.8 | [-2.3; 0.8] | 0.352 |
| *24 hours × N_2_O* | -1.2 | [-2.8; 0.3] | 0.129 |
| *1 week × N_2_O* | -0.8 | [-2.4; 0.7] | 0.31 |
| *2 weeks × N_2_O* | -0.9 | [-2.4; 0.7] | 0.289 |
| *Mania (Young Mania Rating Scale)* | | | |
| *Intercept* | 1.4 | [0.6; 2.1] | <0.001 |
| *Time effect* |  |  |  |
| *2 hours* | -0.4 | [-1.1; 0.2] | 0.213 |
| *24 hours* | -0.3 | [-1; 0.4] | 0.395 |
| *1 week* | -0.4 | [-1.1; 0.2] | 0.213 |
| *2 weeks* | -0.7 | [-1.4; 0] | **0.042** |
| *Group effect, N_2_O* | -1 | [-2; 0.1] | 0.072 |
| *Time × group interactions* |  |  |  |
| *2 hours × N_2_O* | 0.9 | [-0.1; 1.8] | 0.073 |
| *24 hours × N_2_O* | 1.2 | [0.2; 2.1] | **0.019** |
| *1 week × N_2_O* | 1.3 | [0.3; 2.2] | **0.01** |
| *2 weeks × N_2_O* | 1 | [0.1; 2] | **0.035** |
| *Dissociative states (Clinician-Administered Dissociative States Scale)* | | | |
| *Intercept* | 2.7 | [1.5; 4] | <0.001 |
| *Time effect* |  |  |  |
| *2 hours* | -0.5 | [-1.2; 0.2] | 0.202 |
| *24 hours* | -0.6 | [-1.3; 0.2] | 0.145 |
| *1 week* | -0.7 | [-1.4; 0.1] | 0.084 |
| *2 weeks* | -0.3 | [-1; 0.4] | 0.411 |
| *Group effect, N_2_O* | -0.1 | [-1.9; 1.6] | 0.892 |
| *Time × group interactions* |  |  |  |
| *2 hours × N_2_O* | 0.7 | [-0.3; 1.7] | 0.188 |
| *24 hours × N_2_O* | 0.8 | [-0.2; 1.8] | 0.131 |
| *1 week × N_2_O* | 0.3 | [-0.7; 1.3] | 0.543 |
| *2 weeks × N_2_O* | -0.5 | [-1.5; 0.6] | 0.386 |
| *Psychotic disorders (Brief Psychiatric Rating Scale)* | | | |
| *Intercept* | 0.7 | [0.2; 1.2] | 0.012 |
| *Time effect* |  |  |  |
| *2 hours* | -0.2 | [-0.5; 0] | 0.115 |
| *24 hours* | -0.3 | [-0.5; 0] | **0.036** |
| *1 week* | -0.2 | [-0.5; 0] | 0.067 |
| *2 weeks* | -0.3 | [-0.5; 0] | **0.036** |
| *Group effect, N_2_O* | -0.3 | [-1; 0.5] | 0.494 |
| *Time × group interactions* |  |  |  |
| *2 hours × N_2_O* | 0.2 | [-0.1; 0.6] | 0.198 |
| *24 hours × N_2_O* | 0.5 | [0.2; 0.9] | **0.003** |
| *1 week × N_2_O* | 0.5 | [0.2; 0.9] | **0.004** |
| *2 weeks × N_2_O* | 0.3 | [-0.1; 0.7] | 0.099 |

| **Table S4.** Results of the Clinical Global Impression Improvement scale scores. Measurements have been categorized in very much and much better (when the score was 1 or 2) versus minimally better to very much worse (with a score ranking from 3 to 7) | | | | | |
| --- | --- | --- | --- | --- | --- |
| **Clinical Impression (CGI improvement)** | | **N_2_O** |  | **Control** | **p values** |
|  |  | **n (%)** |  | **n (%)** |  |
| 2 hours post exposure | Very much & much better | 3 (10.3 %) |  | 1 (3.4 %) | 0.612^ǂ^ |
|  | Minimally better to very much worse | 26 (89.7 %) |  | 28 (96.6 %) |  |
|  |  |  |  |  |  |
| 24 hours post exposure | Very much & much better | 6 (21.4 %) |  | 3 (10.3 %) | 0.297^ǂ^ |
|  | Minimally better to very much worse | 22 (78.6 %) |  | 26 (89.7 %) |  |
|  |  |  |  |  |  |
| 1 week post exposure | Very much & much better | 9 (30.0 %) |  | 2 (6.9 %) | **0.042**ǂ |
|  | Minimally better to very much worse | 21 (70.0 %) |  | 27 (93.1 %) |  |
|  |  |  |  |  |  |
| 2 weeks post exposure | Very much & much better | 7 (24.1 %) |  | 2 (6.9 %) | 0.144^ǂ^ |
|  | Minimally better to very much worse | 22 (75.9 %) |  | 27 (93.1 %) |  |
|  |  |  |  |  |  |
| ǂ Fisher exact test | | | | | |
