# Supplementary material for: Nitrous oxide for late-life depression with inadequate antidepressant response: a randomised controlled trial
Source: eClinicalMedicine. 2026 Apr 2;94:103860. doi: 10.1016/j.eclinm.2026.103860 (PMC13084366; doi:10.1016/j.eclinm.2026.103860)
Supplement: Clinical Trial Protocol [file mmc2.docx]

**Supplement 1: Clinical trial protocol**

| P MOTOR  CHRU DE TOURS  2 Boulevard Tonnellé  37 044 TOURS cedex 9 | 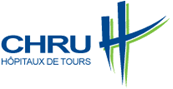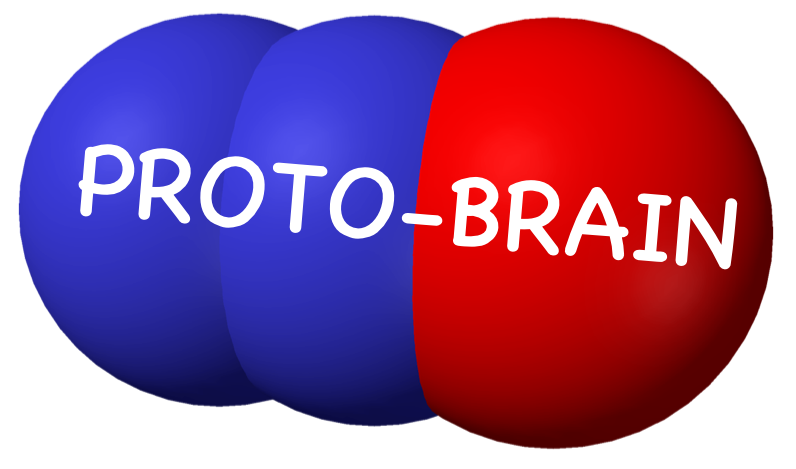 |
| --- | --- |
| **Clinical trial protocol**  **RIPH1 Drug** | |
| STUDY CODE | DR180133 |
| FULL TITLE | Nitrous oxide in Resistant Elderly Depression: a randomized double-blind comparator trial - PROTO-BRAIN |
| COORDINATING INVESTIGATOR | Thomas DESMIDT  CPU-CMRR, CHRU de Tours |
| **PROTOCOL VERSION** | Version n°5.0 |
| PROTOCOL DATE | 24/07/2024 |
| EUDRACT | 2019-004984-31 |
| PPC | Approved on 11/03/2021, by the Comité de Protection des Personnes d du Sud-Ouest et Outre-Mer 4 |
| ANSM | Authorization date: 04/21/2021 |

Substantial Modifications (SM)

| **Version number (after MS)** | **Date** | **MS justification** |
| --- | --- | --- |
| 2.0 | 18/05/2021 | Two new scales added: STAI-Y-A and Subjective VAS |
| 4.0 | 13/02/2023 | Changes to patient visit deadlines  12-month study extension  Addition of a 200 euro ceiling for reimbursement of participants' travel expenses |
| 5.0 | 24/07/2024 | Bringing the protocol into line with European regulation CTR 536/2014  12-month study extension |
|  |  |  |
|  |  |  |

SUMMARY

| **Title** | Nitrous oxide in Resistant Elderly Depression: a randomized double-blind comparator trial: PROTO-BRAIN |
| --- | --- |
| **Version** | Version n°5.0 of 24/07/2024 |
| **Developer** | Tours University Hospital |
| **Investigator Coordinator** | Thomas DESMIDT  CPU-CMRR, CHRU Tours |
| **Background / Justification** | Depression is a severe disorder, not only because of its high prevalence but also because of its limited response rate to conventional antidepressants targeting the aminergic system. Elderly subjects are particularly poor responders to conventional antidepressant treatments. In particular, altered cerebrovascular function, which increases in prevalence with age, has been shown to be one of the main factors in poorer response to antidepressants in the elderly. Recently, products with NMDA receptor antagonist properties, notably ketamine, have shown efficacy in the treatment of resistant depression. However, the benefit/risk balance of this treatment is questionable, due to the frequency and nature of side effects (notably the appearance of psychotic symptoms in more than one in three patients). Another NMDA receptor antagonist, nitrous oxide (N2O, also known as "laughing gas", commonly used in anesthesia), has recently shown antidepressant effects in a group of depressive subjects resistant to conventional treatments, with satisfactory tolerance. Nevertheless, to date, no study has evaluated the efficacy and tolerance of N2O in elderly depression. Given that cerebrovascular factors are implicated in elderly depression, and that N2O has significant effects on cerebrovascular reactivity, reflecting a global cerebral response, we make the general assumption that N2O will be particularly effective in elderly depression, and that cerebrovascular changes induced by N2O exposure will be all the more marked when the antidepressant response is significant. We propose to carry out a double-blind study with a comparator, comparing the efficacy of a Mixture Equimolar Oxygen Nitrous Oxide (MEOPA) as an add-on to conventional antidepressant treatment versus antidepressant treatment plus medical air, on the evolution of depressive symptomatology, in a population of elderly subjects with resistant depression. We propose to evaluate the impact of MEOPA treatment on clinical and cerebrovascular function, using innovative MRI and ultrasound brain imaging techniques. |
| **Main objective** | To compare changes in depressive symptomatology at 2 hours, 24 hours, Week 1, and Week 2 of exposure to MEOPA versus medical air, in a population of elderly subjects with characterized depressive episodes meeting criteria for resistant depression and treated with antidepressants. |
| **Secondary Objectives** | 1. Compare the dynamics of changes in Tissue Pulsatility Imaging (TPI) during gas diffusion between responders (MADRS decrease > 50%), non-responders (MADRS decrease < 50%), and comparator patients. 2. Compare structural (brain volumes, brain leukopathy) and functional (ASL perfusion, BOLD brain pulsatility and connectivity) MRI data between responders, non-responders, and comparator patients at baseline. 3. Compare changes in depressive symptomatology between subjects in the MEOPA group vs. comparator group, as measured by the Hamilton scale for depressive symptom intensity, CGI for change in clinical global impression, QIDS-SR for self-report of mood, STAI-Y-A for anxiety symptoms, and subjective VAS for general health. 4. Compare tolerance between subjects in the MEOPA vs. comparator group, measured in particular by the SSI scale for suicidal ideation, YMRS for manic symptoms, and CADSS and BPRS for dissociative symptoms. |
| **Main judging criterion** | MADRS depression severity scale collected at baseline, 2 hours, 24 hours, Week 1, and Week 2 in MEOPA vs comparator group |
| **Secondary Judging Criteria** | 1. Brain pulsatility indices as measured by ultrasonic IPT imaging 2. Indices of cerebral volumes and white matter lesions measured by MRI, indicators of cerebral atrophy and lesion load; indices of cerebral perfusion measured by ASL-MRI; indices of cerebral pulsatility and connectivity as measured by BOLD signal MRI. 3. A series of scales complementary to the MADRS for the evaluation of depressive symptoms (Hamilton 17-item, CGI – Clinical Global Impression, QIDS-SR – Quick Inventory of Depressive Symptomatology Self-Report, STAI-Y-A – State-Trait Anxiety Inventory, subjective VAS – Visual Analogue Scale) 4. A series of scales to assess possible adverse effects (evolution of scores on the SSI – Scale for Suicidal Ideation, YMRS – Young Mania Rating Scale, CADSS – Clinician Administered Dissociative States Scale, BPRS – Brief Psychiatric Rating Scale) |
| **Study diagram** | Randomized, double-blind, multicenter trial with comparator |
| **Participants** | **Patients over 60 who meet the criteria for a major depressive episode resistant to at least one well-tolerated antidepressant, as assessed by the MGH-ATRQ scale.**  **Inclusion criteria**   - Age between 60 and 90 - Diagnosis of a depressive episode according to DSM-5 criteria, confirmed by the Mini International Neuropsychiatric Interview (MINI) - Montgomery-Åsberg Depression Rating Scale (MADRS) depression score over 20 - Patients resistant to at least one well-tolerated antidepressant for the current depressive episode, as assessed by the MGH-ATRQ scale - Patients able to undergo MEOPA diffusion via a face mask - A person who has signed an informed consent form - Person affiliated to a social security scheme or other scheme   **Non-inclusion criteria**   - Bipolar disorder, schizophrenia, or neurodegenerative disease documented by the MINI and MMSE (non-inclusion if MMSE < 24/30); addiction to one or more drugs - Unstable somatic pathology (especially unstable neurological or cardiological pathologies likely to interfere with MEOPA diffusion), and any unexplained neurological abnormality of recent onset - Presence of significant active psychotic symptoms, at the investigator's discretion - Contraindications to the use of MEOPA and any condition where air is trapped inside the body and its expansion could be dangerous: pneumothorax, emphysema, intestinal obstruction, intracranial hypertension, known and unsubstituted vitamin B12 or B9 deficiency (based on a dosage less than 1 month old), patients requiring oxygen ventilation; any altered state of consciousness preventing patient cooperation; head trauma; gas embolism; diving accident; abdominal gas distension; patients who have recently received an ophthalmic gas (SF6, C3F8, C2F6) used in eye surgery (as long as a gas bubble persists inside the eye and for at least 3 months) - Contraindications to MRI, including claustrophobia, ocular metallic foreign body, pacemaker, neurostimulator, cochlear implants (or non-removable electronic medical equipment), old-generation heart valves, or vascular clips formerly implanted on a cranial aneurysm - Legal incapacity and/or other circumstances rendering the patient unable to understand the nature, purpose, or consequences of the study - A person participating in a clinical drug study or in a period of exclusion from any clinical study due to previous participation |
| **Intervention** | **MEOPA exposure :** The MEOPA exposure procedure will be carried out according to the method detailed and validated in the proof-of-concept article (Nagele et al., 2015). In addition to conventional antidepressant treatment, subjects will receive a mixture of 50% N₂O / 50% O₂ (treatment group) or medical air, i.e. a mixture of 78% N₂ / 22% O₂ (comparator group), for 1 hour. Administration takes place in a suitable facility under continuous medical supervision.  **MRI procedure :** In addition to structural and morphological assessment, signal processing will include measurements of cerebral perfusion and leukoaraiosis load. We will also analyze cerebral pulsatility and connectivity from the BOLD signal.  **Ultrasound procedure (Tissue Pulsatility Imaging – TPI) :** Ultrasound measurement of cerebral pulsatility is similar to transcranial echo-Doppler, except that the ultrasound scanner records the echo-B signal, not the signal from a single artery, but from all movements of the cerebral parenchyma around the middle cerebral artery, with micrometer and millisecond precision, according to a procedure developed by INSERM unit UMR 1253 in Tours and validated in several studies to date. Ultrasound enables continuous, non-invasive, and precise measurement of cerebral pulsatility during MEOPA exposure, to measure variations in cerebrovascular dynamics as the product diffuses. |
| **Course of the study** | **Inclusion visit :** Patients will benefit from an initial visit to the recruiting center for consent signature and inclusion, with pre-treatment clinical evaluation, MRI examination, and vitamin B12 dosage.  **The intervention under study :** Exposure to the study treatment can take place after the inclusion visit and up to 7 days after signing the consent form. Subjects will first be randomized and then exposed to either MEOPA or medical air for 1 hour, with measurements of brain pulsatility by ultrasound (before, during, and after exposure). A new clinical evaluation of the response to treatment will take place 2 hours after the procedure.  **Follow-up visits:**  A follow-up visit will take place the day after the procedure for clinical evaluation and vitamin B12 dosage. Finally, two further visits will take place respectively one week (W1 ±2 days) and two weeks (W2 ±2 days) after exposure to the study treatment. |
| **Number of subjects** | N=60 |
| **number of centers** | 4 (Tours, Rennes, Nice, Nantes) |
| **Search duration** | Inclusion period: 48 months  Mean follow-up time: 21 days ±4 days.  Study period: 61 months. |
| **Expected benefits** | Our study will make it possible to evaluate for the first time the efficacy of MEOPA in the treatment of resistant depression in the elderly. In addition, our study should enable us to further validate imaging biomarkers for predicting response to MEOPA for up to 14 days from the first exposure to MEOPA, in order to considerably reduce the duration of depression, which is known to be a factor in potentially dramatic complications, such as suicide or certain somatic complications, in elderly subjects resistant to conventional antidepressants. The methods used to measure cerebral pulsatility using MRI and ultrasound are innovative, suitable for routine clinical use, and were specifically developed at INSERM UMR 1253, which enjoys international recognition in the field of ultrasound imaging. With this project, we hope to further validate a therapeutic alternative for elderly depression that could rapidly prove effective and have a favorable benefit/risk ratio, as well as to further demonstrate the clinical relevance of brain imaging in improving the management of elderly depressive patients. |

List of abbreviations

| ANR  ANSM | French National Research Agency  Agence Nationale de Sécurité du Médicament et des Produits de Santé (French Agency for the Safety of Medicines and Health Products) |
| --- | --- |
| ARC  ASL  BOLD | Clinical Research Associate  Arterial Spin Labeling  Blood-Oxygen-Level Dependent |
| BPC  BPRS  CADSS  CHRU  CHU  CIC | Good Clinical Practice  Brief Psychiatric Rating Scale  Clinician Administered Dissociative States Scale  Regional University Hospital  University Hospital  Clinical Investigation Center |
| PPC | Comité de Protection des Personnes |
| CNIL | Commission Nationale de l'Informatique et des Libertés (French Data Protection Authority) |
| CRF  DSM  DSUR  ECG | Case Report Form (observation booklet)  Diagnostic and Statistical Manual  Development Safety Update Report  Electrocardiogram |
| EvIG | Serious Adverse Event |
| EIG | Serious adverse effect |
| EIGI  IC | Unexpected Serious Adverse Effect  Confidence interval |
| ICH | International Conference on Harmonization |
| IDE | State-qualified nurse |
| INSERM  MRI  MADRS  MINI  MMSE | French National Institute of Health and Medical Research  Magnetic Resonance Imaging  Montgomery Asberg Depression Rating Scale  Mini International Neuropsychiatric Interview  Mini Mental State Examination |
| MR  N_2_O  NMDA  O_2_  POS  QIDS-SR  SSI | Reference Methodology  Nitrous oxide  N-Methyl-D-Aspartate  Oxygen  Standard operating procedure  Quick Inventory of Depressive Symptomatology Self Report  Scale for Suicidal Ideation |
| SUSAR | Suspected Unexpected Serious Adverse Reaction |
| TEC  PET scan  IPT  YMRS | Clinical Study Technician  Positron Emission Tomography coupled with a scanner  Tissue Pulsatility Imaging  Young Mania Rating Scale |

**CONTENTS**

Table content

[Substantial Modifications (SM) 2](#_Toc201523354)

[SUMMARY 3](#_Toc201523355)

[List of abbreviations 8](#_Toc201523356)

[CONTENTS 10](#_Toc201523357)

[1. Context and rationale 13](#_Toc201523358)

[1.1. Context 13](#_Toc201523359)

[1.1.1. Depression in the elderly, cerebrovascular abnormalities and resistance to treatment 13](#_Toc201523360)

[1.1.2. Nitrous oxide (N_2_O), a new antidepressant specifically adapted to depression in the elderly? 14](#_Toc201523361)

[1.1.3. Towards the identification of biomarkers of response to N_2_O in depression: the use of markers of cerebrovascular dynamics by MRI and Ultrasound 15](#_Toc201523362)

[1.1.4. Originality of the PROTOBRAIN project 16](#_Toc201523363)

[1.2. Benefit/risk balance 16](#_Toc201523364)

[2. Objectives 17](#_Toc201523365)

[2.1. Main objective 17](#_Toc201523366)

[2.2. Secondary objectives 17](#_Toc201523367)

[3. Judging criteria 18](#_Toc201523368)

[3.1. Primary endpoint 18](#_Toc201523369)

[3.2. Secondary endpoints 18](#_Toc201523370)

[4. Research methodology 18](#_Toc201523371)

[4.1 Study diagram 18](#_Toc201523372)

[4.2 Number of subjects required 18](#_Toc201523373)

[4.3 Randomization 19](#_Toc201523374)

[4.4 Blinding 19](#_Toc201523375)

[5. Participants 19](#_Toc201523376)

[5.1 Subject selection and recruitment 19](#_Toc201523377)

[5.2 Subject selection criteria 19](#_Toc201523378)

[5.2.1 Inclusion criteria 20](#_Toc201523379)

[5.2.2 Non-inclusion criteria 20](#_Toc201523380)

[5.3 Exclusion period for participants in other research 20](#_Toc201523381)

[6. Interventions 21](#_Toc201523382)

[6.1 Intervention under study 21](#_Toc201523383)

[6.2 Comparator 21](#_Toc201523384)

[6.3 Modification of the intervention 21](#_Toc201523385)

[6.4 Support for intervention 22](#_Toc201523386)

[6.5 Concurrent treatments and procedures 22](#_Toc201523387)

[6.6 Prohibited concomitant treatments 22](#_Toc201523388)

[7. Course of the study 23](#_Toc201523389)

[7.1 Inclusion and baseline assessment 23](#_Toc201523390)

[7.2 Exposure to study product/Intervention 23](#_Toc201523391)

[7.3 Participant follow-up 24](#_Toc201523392)

[7.4 Study duration 25](#_Toc201523393)

[7.5 Permanent or temporary discontinuation rules 25](#_Toc201523394)

[7.5.1 Discontinuation of a participant’s involvement in the study 25](#_Toc201523395)

[7.5.2 Discontinuation of all or part of the study 25](#_Toc201523396)

[8. Study feasibility 26](#_Toc201523397)

[9. Expected benefits 27](#_Toc201523398)

[10. Data collection and management 27](#_Toc201523399)

[10.1 Data collection 27](#_Toc201523400)

[10.2 Data management 28](#_Toc201523401)

[10.3 Quality control 28](#_Toc201523402)

[10.4 Data access 29](#_Toc201523403)

[10.5 Source data 29](#_Toc201523404)

[10.6 Data confidentiality 29](#_Toc201523405)

[11. Statistical analysis 30](#_Toc201523406)

[11.1 General 30](#_Toc201523407)

[11.2 Definition of analysis populations 30](#_Toc201523408)

[11.3 Description of baseline characteristics 30](#_Toc201523409)

[11.4 Analysis of the primary endpoint 30](#_Toc201523410)

[11.5 Analysis of the secondary endpoints 30](#_Toc201523411)

[12. Safety assessment 31](#_Toc201523412)

[12.1 Investigator's responsibilities 31](#_Toc201523413)

[12.1.1. Reporting serious adverse events 31](#_Toc201523414)

[12.1.2 Assessing the severity and causality of serious adverse events related to the study 31](#_Toc201523415)

[12.1.3 Assessing the severity of the serious adverse events 32](#_Toc201523416)

[12.1.4 Deadlines for notifying the sponsor and means of transmission 32](#_Toc201523417)

[12.1.5 Study-specific provisions 32](#_Toc201523418)

[12.1.6 Notification of non-serious adverse events 33](#_Toc201523419)

[12.2 The sponsor’s responsibilities 33](#_Toc201523420)

[12.2.1 Collection and evaluation adverse events 33](#_Toc201523421)

[12.2.2 Safety reporting to authorities 34](#_Toc201523422)

[12.2.3 Information provided to investigators by the sponsor 36](#_Toc201523423)

[12.3 Oversight committee 36](#_Toc201523424)

[12.4 Follow-up care methods and duration after adverse events 36](#_Toc201523425)

[13. Regulatory and ethical considerations 37](#_Toc201523426)

[13.1 CNIL 37](#_Toc201523427)

[13.2 Ethics Committee (Comité de Protection des Personnes) 37](#_Toc201523428)

[13.3 Substantial modifications 37](#_Toc201523429)

[13.4 Information and consent 37](#_Toc201523430)

[13.5 Biological sample collection 38](#_Toc201523431)

[13.6 Insurance 38](#_Toc201523432)

[13.7 Record-keeping 38](#_Toc201523433)

[13.8 Archiving of documents and data at the end of the study 38](#_Toc201523434)

[14. Publication policy 39](#_Toc201523435)

[14.1 General 39](#_Toc201523436)

[14.2 Authorship 39](#_Toc201523437)

[14.3 Communication of results to study participants 39](#_Toc201523438)

[14.4 Data sharing 39](#_Toc201523439)

[15 Financial aspects 39](#_Toc201523440)

[15.2 Study budget 40](#_Toc201523441)

[15.3 Participant compensation 40](#_Toc201523442)

[Bibliography 41](#_Toc201523443)

[Appendix 43](#_Toc201523444)

1. Context and rationale
   1. Context

### Depression in the elderly, cerebrovascular abnormalities and resistance to treatment

According to the World Health Organization, depressive disorders have become the second leading cause of disability worldwide, and the leading cause in terms of years lived with disability (Whiteford et al., 2013). Depression is indeed a severe disorder, not only because of its high prevalence (lifetime prevalence of 4.4% to 20% in the general population (Bakish, 2001)) but also because a significant proportion of depressed patients are resistant to conventional antidepressants (Cipriani et al., 2009). What’s more, treatment generally requires long-term (several months or years) medication management, and general resistance to treatment contributes significantly to a considerable negative impact in social, economic, and health terms.

A large body of literature now exists on the pathophysiology of depression, revealing a series of changes in the brain biology of patients with major depressive episodes, including abnormalities in neurotransmission, neurogenesis, neuronal activation, and neuroendocrine and inflammatory processes. In the elderly, other brain processes have been specifically implicated. In particular, there is growing evidence that the pathophysiology of depression in the elderly involves abnormalities in cerebrovascular function (Taylor et al., 2013; Aizenstein et al., 2016). In particular, depression in the elderly is consistently associated with a greater lesion load in white matter, visualized on MRI and reflecting abnormalities in small cerebral arteries (Aizenstein et al., 2016). Thus, cerebrovascular lesions often increase with age, under the influence of cardiovascular risk factors such as hypertension, and cerebrovascular dysfunction can induce, promote, or perpetuate a depressive episode. Moreover, the association between depression and cerebrovascular disorders is bidirectional, so that the presence of cerebrovascular abnormalities increases the risk of developing depression, and conversely, depression favours the development of cerebrovascular abnormalities and exposes patients, for example, to an increased risk of stroke, independently of other confounding factors (Pan et al., 2011). Finally, cardio- and cerebrovascular disease is one of the most frequent causes of death in depressed patients (Zivin et al., 2015).

Several pathophysiological hypotheses have been proposed to account for the association between depression and cerebrovascular disease. Most studies find cerebral hypoperfusion in depression, whether by MRI, PET scan, or ultrasound methods (Taylor et al., 2013). Moreover, this hypoperfusion seems to be restored with remission of depressive symptoms and could be a marker of antidepressant resistance. Our team has shown that depression is associated with cerebrovascular abnormalities related to the pulsatility of cerebral micromovements (measured by MRI and ultrasound), suggesting an early disturbance in the dynamics of cerebrovascular changes that could favour cerebral atrophy and the appearance of lesions in small arteries (Desmidt et al., 2011; Desmidt et al., 2017).

Finally, the presence of cerebrovascular abnormalities in depression has been shown to be a significant factor in resistance to antidepressants (Aizenstein et al., 2016). Indeed, conventional antidepressants only partially modify cerebrovascular function and insufficiently restore the cerebrovascular dynamics necessary for remission of depressive symptoms, particularly in depressive patients with significant cerebrovascular abnormalities. Conversely, some studies have shown that the addition of cerebral vasodilators such as nimodipine to conventional antidepressants led to greater symptomatic remission (Taragano et al., 2005). However, certain emerging antidepressants, including nitrous oxide, have effects not only on certain neurotransmitters such as NMDA, but also significantly on cerebral vasoreactivity, and could therefore prove particularly effective in elderly depression. However, to date, no study has specifically evaluated the efficacy of nitrous oxide in elderly depression, whose cerebrovascular abnormalities make it a disorder resistant to conventional antidepressants.

### Nitrous oxide (N_2_O), a new antidepressant specifically adapted to depression in the elderly?

The most widely prescribed conventional antidepressants act by increasing the level of monoaminergic neurotransmitters (serotonin, dopamine, or noradrenaline). However, meta-analyses suggest that these therapeutic agents, which modulate monoaminergic neurotransmission, are only effective as a first-line treatment for around 50% of patients suffering from a characterized depressive episode (Cipriani et al., 2009) and often produce side effects that limit their use.

Recently, products with different brain targets have emerged in the treatment of resistant depression. Phase III trials have demonstrated the efficacy of derivatives of ketamine—an NMDA antagonist used in anesthesia—in resistant depression. Ketamine showed rapid effects on resistant patients, but certain side effects, notably the triggering of psychotic symptoms, may counterbalance its clinical benefits (Chaki, 2017).
Another emerging therapeutic, nitrous oxide (N₂O, also known as "laughing gas", commonly used in anesthesia), recently demonstrated antidepressant effects in a group of middle-aged depressive subjects resistant to conventional treatments (Nagele et al., 2015). In this pilot study, 20 patients with resistant depression were exposed for one hour to a 50% N₂O / 50% O₂ mixture (Mélange Équimolaire Oxygène Protoxyde d’Azote – MEOPA) or to a 50% nitrogen / 50% O₂ mixture, the latter representing the placebo treatment. A significant improvement in depressive symptoms was observed at 2 hours, 24 hours, and for some patients at 1 week after exposure to N₂O, compared with placebo (at 24 hours: 5.5 points, 95% CI = 2.5–8.5, better than placebo, on the Hamilton scale). There were no severe side effects, and the adverse events noted were brief and of low intensity.

In addition to NMDA receptors, N₂O has numerous brain targets which may account for its clinical efficacy in depression. In particular, N₂O has marked cerebrovascular effects, including a vasodilatory effect, which may be the overall consequence of N₂O’s effect on the brain. A study on healthy subjects using the MRI technique of ASL (Arterial Spin Labelling) showed a very significant vasodilatory effect, as well as a decrease in oxygen extraction, upon exposure to a low dose of N₂O (Dashdorj et al., 2013). However, while the benefits seem promising—because they are rapid, sustained, and associated with low side effects—the mechanisms underlying the antidepressant response to N₂O are poorly understood. To date, there have been no studies of elderly depressive patients evaluating the cerebrovascular response to N₂O. Given that cerebrovascular factors are widely implicated in elderly depression, and that N₂O has highly significant effects on cerebrovascular reactivity, reflecting a global cerebral response, we make the general assumption that N₂O will be particularly effective in elderly depression, and that cerebrovascular changes in response to N₂O exposure will be all the more marked as the antidepressant response is significant.

### Towards the identification of biomarkers of response to N_2_O in depression: the use of markers of cerebrovascular dynamics by MRI and Ultrasound

Predicting response to treatment in depression to increase the chances of a favorable outcome and reduce the impact and duration of depression is a major challenge for clinicians. Some predictive factors—whether clinical, cognitive, physiological, or imaging-based—have been studied in depression, but data are lacking to recommend their use in routine clinical practice.

Recent evidence suggests that neuroimaging biomarkers may be reliable predictors of treatment response in depression (Aizenstein et al., 2014). MRI techniques have been shown to inform the treatment response profile. For example, the presence of certain markers of cerebrovascular lesions leads to the use of higher doses of antidepressants or specific drug classes. Functional MRI techniques can also be used to measure early synaptic changes associated with antidepressant exposure, and the team in Pittsburgh, USA, with whom we have a partnership, recently showed that changes in brain connectivity in depressed patients after the first dose of antidepressant could predict treatment response at 3 months—whereas it usually takes 3 to 4 weeks of treatment before the first clinical improvements are observed (Karim et al., 2017).

Beyond synaptic activity, brain MRI can now measure physiological fluctuations linked to brain movement and pulsatility, secondary to cerebrovascular reactivity. This method is based on the measurement of BOLD activity, and its signal processing was developed by a team in Toronto, Canada (Makedonov et al., 2016), then validated in patients with cerebrovascular lesions and Alzheimer’s disease.

However, MRI is a relatively expensive tool, and access to it in routine clinical practice is not guaranteed. Less costly and more accessible alternatives for predicting treatment response in depression have been suggested. In particular, our team has developed an ultrasound technique for measuring cerebral pulsatility and the amplitudes of cerebral micromovements (TPI – Tissue Pulsatility Imaging), linked to cerebrovascular reactivity, which we have shown to be disrupted in depression (Desmidt et al., 2011; Desmidt et al., 2017).

TPI has been validated in healthy subjects using hyperventilation tests (Kucewicz et al., 2008) and visual tasks (Kucewicz et al., 2007), and our team has further validated it in clinical settings—in patients with cerebrovascular lesions (Ternifi et al., 2014), in patients with orthostatic hypotension (Biogeau et al., 2017), and in two studies on depressed patients (Desmidt et al., 2011; Desmidt et al., 2017). We have thus shown that the amplitude of brain movements, measured with micrometer precision, was reduced in elderly depressed subjects with cardiovascular disease and in subjects with cerebrovascular lesions visualized on MRI. Even more recently, we have shown that brain pulsatility was increased in middle-aged depressed subjects with no apparent cerebrovascular lesions on MRI, suggesting that depression is associated with disturbances in cerebrovascular reactivity even before the onset of irreversible brain lesions.

We hypothesize that TPI, a sophisticated, non-invasive, inexpensive tool that can be used continuously at the patient's bedside, could complement MRI in characterizing the cerebrovascular physiology of the N₂O response in depression—particularly because N₂O has significant cerebral vasodilatory properties, and this short-term vasodilation could predict long-term antidepressant response.

### Originality of the PROTOBRAIN project

To date, only one pilot study has assessed the efficacy and safety of MEOPA in depression (Nagele et al., 2015). No study has tested MEOPA in a population of elderly depressed subjects, who are particularly exposed to the risk of treatment resistance, due in particular to the significant involvement of cerebrovascular factors. Our study would thus be the first to assess the efficacy and tolerability of MEOPA in elderly depression. We also plan to implement innovative, high-performance neuroimaging techniques in ultrasound and MRI, developed by INSERM UMR 1253 in Tours and available to the HUGOPSY network, which could prove to be reliable and relevant markers of response to MEOPA in elderly depression.

- 1. Benefit/risk balance

MEOPA exposure will be the same as in the pilot study by Nagele et al., 2015, i.e. a one-hour exposure to an equimolar mixture of oxygen and nitrous oxide. In this randomized, double-blind, placebo-controlled pilot study of 20 patients with drug-resistant depression, depressive symptoms were significantly improved (Hamilton scale) at 2 h and 24 h after MEOPA administration. The results of this preliminary study therefore support the efficacy of MEOPA in patients with drug-resistant depression.

No serious adverse events were observed in this study; only moderate and transient non-serious adverse events were reported (nausea/vomiting, headache, anxiety, dizziness, etc.).
In general, mild and transient adverse effects such as digestive and neurological disorders (paresthesia, agitation, anxiety, dizziness, headaches) have been reported with MEOPA during occasional use, as in dentistry and obstetrics. Participants in this study will therefore be monitored throughout MEOPA administration and for at least 2 hours afterwards. Other MEOPA toxicities, such as myeloneuropathy and megaloblastic anemia (due to inactivation of vitamin B12 by oxidation of its cobalt atom), have only been reported in cases of repeated and prolonged administration, which is not the case here. Nevertheless, as subclinical vitamin B12 deficiency cannot be formally ruled out in the absence of a functional deficiency, a vitamin B12 assay is planned for participants in this study before and after MEOPA administration.

The only constraint will be the time required for imaging examinations (MRI and ultrasound), which otherwise pose no particular risk, as these are totally non-invasive diagnostic procedures.

The benefit-risk balance does not appear to pose any particular concern and may be considered neutral, insofar as the investigations carried out are non-invasive and do not result in any change in patient management—except potentially favorable changes should this treatment prove to be an effective therapeutic alternative.

1. Objectives
   1. Main objective

To compare changes in depressive symptomatology at 2 hours, 24 hours, Week 1, and Week 2 of exposure to MEOPA versus comparator, in a population of elderly subjects with characterized depressive episodes meeting criteria for resistant depression and treated with antidepressants. According to the standards of double-blind comparator studies testing the efficacy of antidepressants, the primary endpoint will be the score on the MADRS depressive symptomatology intensity scale.

- 1. Secondary objectives

1. Compare the dynamics of changes in Tissue Pulsatility Imaging (TPI) during gas diffusion between responders (MADRS decrease > 50%), non-responders (MADRS decrease < 50%), and comparator patients.
2. Compare structural (brain volumes, cerebral leukopathy) and functional (ASL perfusion, BOLD brain pulsatility and connectivity) MRI data between responders, non-responders, and comparator patients.
3. Compare changes in depressive symptomatology between subjects in the MEOPA vs. comparator group, as measured by the Hamilton scale for intensity of depressive symptoms, CGI for change in clinical global impression, QIDS-SR for self-report of mood, STAI-Y-A for anxiety symptoms, and subjective VAS for general health.
4. Compare tolerance between subjects in the MEOPA vs. comparator group, measured in particular by the SSI scale for suicidal ideation, YMRS for manic symptoms, and CADSS and BPRS for dissociative symptoms.
5. Judging criteria
   1. Primary endpoint

MADRS depression severity scale collected at baseline, 2 hours, 24 hours, Week 1, and Week 2 after MEOPA exposure.

- 1. Secondary endpoints

1. Indices of cerebral pulsatility as measured by ultrasonic IPT imaging.

2. Indices of cerebral volumes and white matter lesions measured by MRI, as indicators of cerebral atrophy and lesion load; indices of cerebral perfusion measured by ASL-MRI; indices of cerebral pulsatility and connectivity as measured by BOLD-signal MRI.

3. A series of scales complementary to the MADRS for assessing the evolution of depressive symptoms (Hamilton 17-item, CGI – Clinical Global Impression, QIDS-SR – Quick Inventory of Depressive Symptomatology Self-Report, STAI-Y-A – State-Trait Anxiety Inventory, and subjective VAS – Visual Analogue Scale).

4. A series of scales to assess possible adverse effects (evolution of scores on the SSI – Scale for Suicidal Ideation, YMRS – Young Mania Rating Scale, CADSS – Clinician-Administered Dissociative States Scale, and BPRS – Brief Psychiatric Rating Scale)

4.  Research methodology

- 1. Study design

Randomized, double-blind, multicenter trial with comparator.

- 1. Number of subjects required

According to the literature (Nagele et al., 2015), we can postulate the following:

- Expected mean change of -5.5 points for the MEOPA group
- Expected mean change of -2.8 points for the comparator group
- Standard deviation of 5 points

This leads us to include 73 patients per group, considering a power of 90% and an alpha risk of 5%. Taking into account the fact that there is one baseline and four follow-up data points (2 h, 24 h, Week 1, and Week 2), and assuming a correlation of 0.5 between baseline and follow-up measurements (a low correlation value, conservative assumption), we need to include 28 patients per group (Borm and Vickers approach; Borm et al., 2007; Vickers, 2003).
Since randomization is stratified by center (4 participating centers), we plan to include 60 subjects, 15 per center.

- 1. Randomization

The randomization list will be generated by a biostatistician from CIC INSERM 1415 at CHRU de Tours, using a 1:1 ratio. This randomization will be stratified according to the investigating center. The list will then be implemented in a software program accessible via the Internet (Ennov Clinical®). Patient randomization can therefore be carried out in real time, 24 hours a day. This randomization will take place just before the administration of MEOPA or the comparator.

- 1. Blinding

The psychiatrist-investigator will carry out the protocol assessments blinded to the outcome of the randomization. As the equipment used for MEOPA/medical air exposure is not identical and cannot be standardized, it will be hidden behind a screen so that the patient is also blinded. On the other hand, the qualified personnel administering the treatment will not be blinded. Finally, at the very end of visit Week 2 (W2), at the end of the protocol, we will assess whether patients believed they had identified their group (MEOPA or medical air), and whether they felt they had received MEOPA or not.

1. Participants
   1. Subject selection and recruitment

The four centers involved in this project have recognized expertise in the field of depression in the elderly, and the project’s investigators, as part of their main clinical activity, provide consultations for elderly patients with characterized depressive episodes, many of whom have already received a series of antidepressant treatments. Subjects will be selected and recruited by these expert investigators, either on an outpatient or inpatient basis. The investigators will propose that elderly patients with resistant depression take part in the PROTO-BRAIN protocol and will provide them with an information letter. At least 48 hours must elapse before inclusion.
Given the volume of active patients (over 60 consultations per month in the geriatric psychiatry unit in Tours, for example, which is also an expert center for resistant depression), the objective of including one patient every two months in each center seems reasonable.

- 1. Subject selection criteria
     1. Inclusion criteria
- Age between 60 and 90.
- Diagnosis of a depressive episode according to DSM-5 criteria, confirmed by the Mini International Neuropsychiatric Interview (MINI).
- Montgomery-Åsberg Depression Rating Scale (MADRS) score greater than 20.
- Resistance to at least one well-tolerated antidepressant for the current depressive episode, as assessed by the MGH-ATRQ scale.
- Ability to undergo MEOPA administration via a face mask.
- Signed informed consent form.
- Affiliation with a social security scheme or equivalent coverage.
  - 1. Non-inclusion criteria
- Bipolar disorder, schizophrenia, or neurodegenerative disease documented by the MINI and MMSE (non-inclusion if MMSE < 24/30); addiction to one or more substances.
- Unstable somatic pathology (especially unstable neurological or cardiological conditions likely to interfere with MEOPA administration) and any unexplained neurological abnormality of recent onset.
- Presence of significant active psychotic symptoms, at the investigator's discretion.
- Contraindications to the use of MEOPA and any condition where air is trapped inside the body and its expansion could be dangerous: pneumothorax, emphysema, intestinal obstruction, intracranial hypertension, known and untreated vitamin B12 or B9 deficiency (based on an assay less than one month old); patients requiring pure oxygen ventilation; any altered state of consciousness preventing patient cooperation; head trauma; gas embolism; diving accident; abdominal gas distension; patients who have recently received an ophthalmic gas (SF6, C3F8, C2F6) used in eye surgery (as long as a gas bubble persists inside the eye and for at least 3 months).
- Contraindications to MRI, including claustrophobia, ocular metallic foreign body, pacemaker, neurostimulator, cochlear implants (or non-removable electronic medical equipment), old-generation heart valves, or vascular clips formerly implanted on a cranial aneurysm.
- Legal incapacity and/or other circumstances rendering the patient unable to understand the nature, purpose, or consequences of the study.
- Participation in another clinical drug study or being in a period of exclusion following previous participation in a clinical study.
  1. Exclusion period for participants in other research

In order to avoid any risk of drug interactions, research subjects may not be included in another therapeutic study for the duration of their participation in the study. They may, however, participate in an observational study (subject to the coordinator’s approval). No exclusion period is required after this study.

1. Interventions
   1. Intervention under study

**MEOPA exposure :** The MEOPA exposure procedure will be carried out according to the method detailed and validated in the proof-of-concept article (Nagele et al., 2015). In addition to conventional antidepressant treatment, subjects in the treatment group will receive a mixture of 50% N₂O / 50% O₂ for 1 hour. Administration will take place in a suitable facility under continuous medical supervision by specifically trained personnel. The mixture will be administered via a standard mask. The rate of administration will be approximately 6–9 liters per minute. To ensure the safety of participants included in the trial, administration of the mixture will be immediately discontinued in the event of loss of verbal contact (in accordance with section 4.2 of the Kalinox RCP). After exposure to N₂O, subjects will receive oxygen by mask for 15 minutes to avoid hypoxia, with continued monitoring. Monitoring will be continuous, with ECG, oximetry, blood pressure, and respiratory measurements during administration of the study treatment, and will continue after the end of the procedure for as long as necessary. Finally, discharge from the investigating center will be at the physician's discretion, following a final clinical evaluation.

In the event of prolonged exposure, vitamin B12 deficiency may be observed, leading to neuropathy-type adverse effects. Plasma measurement of vitamin B12 before and after treatment will enable monitoring of the risk of vitamin B12 depletion (note that none of the depressed patients in the N₂O proof-of-concept study showed abnormalities in vitamin B12 metabolism).

- 1. Comparator

Subjects in the comparator group will be exposed to an identical procedure to those in the treatment group, with the exception of the product used, which will be medical air, i.e. a mixture of 78% N₂ / 22% O₂. Nitrogen and oxygen are the two most common gases in atmospheric air and are not considered to have significant cerebrovascular effects at these concentrations.
Furthermore, a study (Dashdorj et al., 2013) compared the effects on cerebral perfusion and metabolism between medical air, medical air + 40% O₂, and medical air + 40% O₂ + 30% N₂O, and showed that there was no difference in effects between medical air alone and medical air + 40% O₂, whereas the gas mixture containing N₂O had a significantly greater cerebrovascular effect than the other two gaseous compounds.

- 1. Modification of the intervention

It is possible that some subjects may not complete the full hour of exposure to the product. In the pilot study (Nagele et al., 2015), only two subjects discontinued MEOPA exposure early due to discomfort or nausea/vomiting. In the event of premature discontinuation of the procedure, this information will be recorded in the observation notebook, and the subject will continue the study in accordance with the protocol until the end of follow-up (up to W2), following the same study procedures.

- 1. Support for intervention

According to literature data (van Amsterdam et al., 2015) and the results of the pilot study (Nagele et al., 2015), MEOPA is generally well tolerated and accepted by subjects, even during prolonged one-hour exposures. Subjects will be supervised throughout the protocol by professionals specialized in drug trials, in a secure medical environment, which should, in particular, reassure depressive subjects under the care of psychiatrists with expertise in late-life depression and facilitate their adherence.

- 1. Concurrent treatments and procedures

**Conventional antidepressant treatment :** At study inclusion, all subjects must have been taking a conventional antidepressant (Selective Serotonin Reuptake Inhibitor, Serotonin and Noradrenaline Reuptake Inhibitor, etc.) for at least 8 weeks, at an effective dose, as part of their routine care and prior to the protocol, in order to be considered resistant to at least one well-taken antidepressant. The same antidepressant will be continued by the patient for the duration of the protocol (14 days). It should be noted that there is a risk of MEOPA potentiating the hypnotic effects of centrally acting drugs (including antidepressants).

**MRI procedure :** In addition to structural and morphological assessment, signal processing will include measurements of cerebral perfusion and leukoaraiosis load (using the method described by Wen et al. – Wen et al., 2004). We will also analyze cerebral pulsatility from the BOLD signal using the method described by our colleagues at the University of Toronto (Makedonov et al., 2013).

**Ultrasound procedure (Tissue Pulsatility Imaging – TPI) :** Ultrasound measurement of cerebral pulsatility is similar to transcranial echo-Doppler, except that the ultrasound scanner records the Echo-B signal, not just the signal from a single artery, but from all movements of the cerebral parenchyma around the middle cerebral artery, with micrometer and millisecond precision, using a procedure developed by INSERM UMR 1253 and validated in several studies to date. Ultrasound enables continuous, non-invasive, precise measurement of cerebral pulsatility during exposure to MEOPA or comparator, to measure variations in cerebrovascular dynamics as the product diffuses.

- 1. Prohibited concomitant treatments

Any recent treatment with an ophthalmic gas (SF₆, C₃F₈, C₂F₆) within the past 3 months (in accordance with sections 4.3 and 4.5 of the Kalinox SPC) will preclude participation in this clinical study.

1. Course of the study
   1. Inclusion and baseline assessment

Subjects will have a minimum 48-hour cooling-off period between information and inclusion. At inclusion, subjects will attend a first visit to the recruiting center for consent signature and study enrollment. During this first visit, the following tests will be performed:

- **Clinical and psychometric evaluation** : demographic data, medical and surgical history summarized using the Cumulative Illness Rating Scale (CIRS) score; level of resistance to the depressive episode (measured by the Maudsley Staging Method); treatments; psychometric scales including MADRS, MINI, MGH-ATRQ, MMSE, and all scales assessing depressive symptoms (Hamilton – to assess the intensity of depressive symptoms; CGI – for change in clinical global impression; QIDS-SR – for mood self-evaluation; STAI-Y-A – for anxiety symptoms; subjective VAS – for general health status) and adverse side effects (modified SSI – for suicidal ideation; YMRS – for manic symptoms; CADSS – for dissociative symptoms; BPRS – for psychotic symptoms).
- **The clinical examination** at the inclusion visit will include a complete neurological examination to detect localized neurological deficits, a cardiological and pulmonary examination to detect signs of cardiovascular or respiratory pathologies such as heart failure or emphysema, and vital signs (blood pressure, temperature, oxygen saturation) to detect signs of infection, arterial hypertension, and hypoxia in particular.
- **Neuroimaging evaluation** : MRI and IPT (approximately 10 minutes before exposure to the study product)
- **A blood test** for vitamins B12 and B9 (a test less than 1 month old may be used to validate the non-inclusion criterion).
  1. Exposure to study product/Intervention

Exposure to the MEOPA study product can take place on the day of inclusion and up to one week later.

Randomization and exposure to MEOPA or medical air will follow the procedure described in the proof-of-concept article on the use of MEOPA in depression (Nagele et al., 2015), with TPI measurements taken 10 minutes before, during exposure, and 10 minutes after completion.

A caregiver must be present at the participant's home for 24 hours after gas inhalation to monitor the situation, with access to a 24-hour telephone hotline, the number of which will be provided at the time of enrollment.

Depending on the assessment of suicidal risk, in the event of a score above 9 on the SSI scale, an assessment by a psychiatrist (who may be the study investigator) will be required. Management will include, at a minimum, systematic notification of the treating psychiatrist and attending physician, and the organization of follow-up beyond the 2-week protocol if not already planned.

- 1. Participant follow-up

Two hours after exposure to treatment, an initial follow-up clinical evaluation will take place. A new clinical evaluation will be carried out the following day, 24 hours after exposure to treatment, including a blood sample for a vitamin B12 assay.

Two further visits will take place at Week 1 (W1 ± 2 days) and Week 2 (W2 ± 2 days) intervals from the study intervention for clinical assessment of depressive symptoms and tolerance.

If an abnormality is detected in the vitamin B12 assay, treatment will be offered in accordance with standard care procedures (additional investigations, supplementation, etc.).

At the end of the assays, the remaining blood samples from patients at the Tours center only will be used to build up a collection, which will be kept indefinitely at the Tours University Hospital for further research on the same theme.

**Table 1 - PROTO-BRAIN study visits**

|  | Screening visit | Inclusion  D0 | Exposure to study treatment (D0 ±7 days) | D1 | W1 | W2 |
| --- | --- | --- | --- | --- | --- | --- |
| Information | X |  |  |  |  |  |
| Signature of consent |  | X |  |  |  |  |
| Randomization |  | X |  |  |  |  |
| Clinical and psychometric* assessment |  | Baseline X | X  (after 2 hours) | X | X | X |
| MRI |  | X |  |  |  |  |
| IPT |  |  | XXX  before, during, after |  |  |  |
| Exposure to N_2_O or -Comparator |  |  | X |  |  |  |
| Dosage of vitamins B12 and B9 |  | X** |  | X |  |  |
| AR collection |  | X | X | X | X | X |

** psychometric scales and all scales assessing depressive symptoms and side effects: MADRS, MINI, MGH-ATRQ, MMSE, Hamilton, CGI, QIDS-SR, STAI-Y-A, subjective VAS, SSI-modified, YMRS, CADSS, BPRS*

*** if no dosage available less than 1 month old*

- 1. Study duration

The patient's total duration of participation in the study is 21 days ± 4 days, from the date of inclusion to the date of the last visit.

The inclusion period is 48 months.

The total duration of the study (inclusion period + follow-up) is estimated at 61 months.

As soon as the study is initiated for the first time, the sponsor must inform the competent authority and the CPP without delay of the effective start date of the study (effective start date = date on which the consent form is signed by the first person to take part in the research).

The sponsor will notify the ANSM and the CPP, via the CTIS, of the study end date within 15 days. The research end date corresponds to the end of the participation of the last person to take part in the research or, where applicable, to the end date defined in the protocol.

- 1. Permanent or temporary discontinuation rules
     1. Discontinuation of a participant’s involvement in the study

Subjects may withdraw their consent and request to stop the study at any time and for any reason. The investigator must document the reasons as fully as possible.

The investigator may temporarily or permanently discontinue study treatment for any reason that is in the subject's best interest, particularly in the event of serious adverse events.

If a subject is lost to follow-up, the investigator will make every effort to recontact the person and will indicate in the patient's medical record the actions taken.

- - 1. Discontinuation of all or part of the study

The study may be temporarily or permanently interrupted at any time by the sponsor, in particular on the basis of information provided by the Independent Monitoring Committee (IMC). The IMC will meet by teleconference in the event of a serious unexpected adverse event (SAE/SUSAR), once a year before the safety report (DSUR) is sent to the authorities, or if new data could lead to a reassessment of the study's benefit/risk balance. Similarly, unforeseen events or new information relating to the device, in light of which the objectives of the study or clinical program are unlikely to be achieved, may lead the sponsor to interrupt the study prematurely. The CHRU de Tours reserves the right to discontinue the study at any time if it becomes apparent that the inclusion objectives are not being met.

If the study is stopped prematurely, the sponsor will inform the ANSM and the CPP within 15 days.

1. Study feasibility

The four centers involved in the project all have recognized expertise in the field of treatment-resistant depression in the elderly and neuroimaging, and are regularly engaged in research projects in these areas. We are already collaborating on PHRC-funded projects investigating the links between late-life depression and Alzheimer’s disease through neuroimaging biomarkers (ASAP protocol), apathy in elderly depression (ACTIDEP project), and characterization of cerebral white matter fibers using MRI and histological sections (FIBRATLAS protocol). Furthermore, elderly patients with depression represent a significant proportion of the active case load for recruiting investigators, and the target of enrolling one to two patients every two months per center appears reasonable and readily achievable.

The use of MEOPA and medical air requires standard anesthesia equipment, hyperbaric gas cylinders, high-pressure masks, and continuous vital sign monitoring systems—expertise that can be provided by the investigators and Clinical Investigation Center staff where involved (site authorization will be required in such cases). A feasibility assessment will be conducted at each site. Personnel administering MEOPA or medical air will receive specific training for the procedure.

MRI platforms are available in all participating centers, with image post-processing centralized at the CHU in Tours. Ultrasound measurement of cerebral pulsatility will be performed by qualified staff trained in transcranial Doppler and specifically in the IPT (Tissue Pulsatility Imaging) technique. The centers in Tours, Rennes, and Nice will have their own equipment and technicians. Tours-based technicians will travel to Nantes with portable ultrasound equipment to perform cerebral pulsatility measurements when participants are enrolled there.

The PROTO-BRAIN project builds upon work previously conducted by the INSERM UMR 1253 unit in Tours (Imagerie et Cerveau), notably supported by the ANR (COSTUM and EMPHILINE projects), which led to the development and validation of the Ultrasound Brain Pulse method. These studies demonstrated that cerebrovascular reactivity and Brain Pulse vary with age (COSTUM) and in depression (EMPHILINE). The coordinating investigator also acquired expertise in measuring cerebral pulsatility via MRI at the Geriatric Psychiatry Neuroimaging Lab at the University of Pittsburgh (USA), a method that could complement the ultrasound approach developed by INSERM UMR 1253. A feasibility study for implementing both MRI and ultrasound-based cerebral pulsatility measurements in middle-aged depressive patients exposed to MEOPA is currently underway at the CHU de Tours (Principal Investigator: Dr. Thomas Desmidt, funded by Fondation Th. & R. Planiol and Fondation de l’Avenir, 2017), which will provide the Tours coordinating center with procedural validation prior to launching the PROTO-BRAIN project.

Lastly, the project is supported by CIC 1415, a well-established partner of the investigating team in Tours. CIC-IT is responsible for overall coordination, regulatory oversight, quality control, and processing of MRI and ultrasound data, leveraging its imaging expertise. CIC-P will contribute methodological supervision, biostatistics, investigation management, and data handling.

1. Expected benefits

A significant proportion of depressed elderly subjects are resistant to conventional treatments, which generally involve long-term medication (several months or years), and general resistance to treatment contributes significantly to a considerable negative social, economic and health impact. The development of new, rapidly effective therapies is one of the major challenges in psychiatry to reduce the negative impact of depression. Ketamine is one of these emerging therapies, but its side-effects limit its use in routine clinical practice. N₂O is a potential alternative to ketamine, with better tolerability and few dissociative effects. Our study will be the first to evaluate the efficacy of MEOPA in the treatment of resistant depression in the elderly. In addition, our study should enable further validation of imaging biomarkers that can predict response to MEOPA for up to 14 days, from the first exposure to MEOPA, to considerably reduce the duration of depression, which is known to be a factor in potentially dramatic complications, such as suicide or certain somatic complications, in elderly subjects resistant to conventional antidepressants. The methods used to measure cerebral pulsatility using MRI and ultrasound are innovative and have been specifically developed at INSERM UMR 1253, which enjoys international recognition in the field of ultrasound imaging. With this project, we hope to further validate a therapeutic alternative for elderly depression that could rapidly prove effective, have a favorable benefit/risk ratio and be routinely used in psychiatry, as well as to further demonstrate the clinical relevance of brain imaging to improve the management of elderly depressive patients.

1. Data collection and management
   1. Data collection

A web-based data collection medium will be used for this study. All the information required by the protocol will be collected in this electronic notebook, with the exception of the MRI and ultrasound analyses, which will be stored on a dedicated server for post-processing. The data circuit will be ensured by the use of a unique identifier for each subject, in order to reconcile the databases for statistical analysis. The electronic observation book requires only an Internet connection and a browser. Investigators will be provided with a document to help them use this tool.

- 1. Data management

Study data management will be handled by a data manager from CIC INSERM 1415. The electronic case report form (eCRF) will be developed using Ennov Clinical® software. Data management will follow the standard operating procedures (SOP) in force at CIC INSERM 1415. The clinical research associate (CRA) in charge of the study at the sponsor's level will be trained in the use of the eCRF, and will then be responsible for training investigators and TECs.

Data will be entered into the investigating center via a secure website, and monitored by the CRA, according to the grade and monitoring plan defined for the risk involved in the study. Queries will be edited by the data manager according to a consistency control plan established when the case report is designed.

A review of the data will be carried out before the database is frozen. The database will be frozen according to the SOP in force at CIC INSERM 1415, and the data will be extracted in the format required for statistical analysis.

- 1. Quality control

A Clinical Research Associate (CRA) appointed by the sponsor will ensure that the study is carried out properly, and that the data generated are documented, recorded, and reported, in accordance with the Standard Operating Procedures implemented at the CHRU de Tours and in compliance with Good Clinical Practice and current legislation and regulations.
The investigator and members of **their** team agree to make themselves available for Quality Control visits carried out at regular intervals by the Clinical Research Associate. During these visits, the following elements will be reviewed:

- informed consent
- compliance with the study protocol and procedures defined therein
- quality of data collected in the observation book: accuracy, missing data, consistency of data with "source" documents (medical records, questionnaires, original laboratory results, etc.)
- management of any products
- collection and reporting of Serious Adverse Events

Investigators also agree to accept quality assurance audits by the sponsor and inspections by the competent authorities. All data, all documents and reports may be subject to regulatory audits and inspections, without prejudice to medical confidentiality.

- 1. Data access

In accordance with GCP:

- The sponsor is responsible for obtaining the agreement of all parties involved in the research to guarantee direct access to all research sites, source data, source documents, and reports for quality control and audit purposes by the sponsor.
- Investigators will make available to those responsible for monitoring, quality control, or auditing biomedical research the documents and individual data strictly necessary for this control, in accordance with the legislative and regulatory provisions in force (European Regulation No. 536/2014 of April 16, 2014, relating to clinical trials).
  1. Source data

Source documents are defined as any original document or object used to prove the existence or accuracy of any data or fact recorded during the clinical study.

For the purposes of this study, the source documents include: the patient's medical record, reports of examinations performed, clinical and psychometric evaluation questionnaires, as well as MRI and ultrasound recordings and analyses.

- 1. Data confidentiality

In accordance with the provisions concerning the confidentiality of data to which those responsible for the quality control of biomedical research have access (European Regulation No. 536/2014 of April 16, 2014 relating to clinical trials), persons with direct access will take all necessary precautions to ensure the confidentiality of information relating to the investigational medicinal products, the trials, and the individuals involved, particularly with regard to their identity and the results obtained. These persons, like the investigators themselves, are bound by professional secrecy (under the conditions defined by Articles 226-13 and 226-14 of the French Penal Code).

During or at the end of **the** biomedical research, data collected on subjects and transmitted to the sponsor by the investigators (or any other specialist) will be coded in compliance with **confidentiality rules**. Under no circumstances may the names or addresses of the persons concerned appear in plain text. Only the first letter of the subject's last name and the first letter of their first name will be recorded, together with a coded number specific to the study, indicating the order of subject inclusion.

The sponsor will ensure that each person taking part in the research has given **his or her** written consent for access to individual data concerning **him or her**, strictly limited to what is necessary for the quality control of the research.

Data collected as part of the research will be processed in compliance with current regulations on the protection of personal data, also known as the General Data Protection Regulation (GDPR).

1. Statistical analysis
   1. General

Analyses will be performed using SAS version 9.2 (or later) and/or R software. Statistical analysis will be carried out according to a pre-established statistical analysis plan.

No interim analysis will be carried out.

A statistical analysis report will be drawn up incorporating all the elements to be reported.

A flow diagram will be drawn up.

All statistical tests will be performed at the 5% significance level.

- 1. Definition of analysis populations

All randomized patients will be included in the analyses according to the arm in which they were randomized, whatever treatment they actually received and whatever their fate in the study, according to the principle of intention-to-treat analysis.

- 1. Description of baseline characteristics

The groups resulting from randomization will be described and compared using the following descriptive statistics (no statistical tests will be performed) : for qualitative variables, numbers and percentages; for quantitative variables, mean and standard deviation, or median and interquartile range according to distribution.

- 1. Analysis of the primary endpoint

Changes from baseline in MADRS scores at 2h, 24h, W1 and W2 will be compared between MEOPA and comparator subjects using a mixed model for repeated data.

- 1. Analysis of the secondary endpoints
  2. Changes in brain pulsatility will be compared between responders, non-responders, and comparator subjects using mixed-effects models for repeated measures.
  3. Cerebrovascular burden (leukoaraiosis), cerebral perfusion on ASL, and cerebral pulsatility on BOLD MRI at baseline will be compared between responders, non-responders, and comparator subjects using analysis of variance (ANOVA) models.
  4. Changes in depressive symptomatology between subjects in the MEOPA group and those in the comparator group will be compared using mixed-effects models for repeated measures.
  5. Tolerance between subjects in the MEOPA group and those in the comparator group will be described using descriptive statistics and compared using mixed-effects models for repeated measures.

1. Safety assessment

The definitions used in this paragraph are given in Appendix 1.

- 1. Investigator's responsibilities

12.1.1. Reporting serious adverse events

The investigator notifies the sponsor of all serious adverse events occurring in the course of the research without undue delay and no later than 24 hours after becoming aware of the event, except for those identified in the protocol as not requiring immediate notification. Adverse events also include medication errors and uses not foreseen in the protocol, including misuse or abuse of the drug.

The investigator also notifies the sponsor of adverse events and/or abnormal test results defined in the protocol as crucial to the evaluation of the safety of clinical trial subjects, in accordance with the procedures and deadlines specified in the protocol and in compliance with Good Clinical Practice.

This notification is made via a written report using a specific research notification form, which may be followed by further detailed written reports.

The investigator documents the serious adverse event to the best of his ability and will, if possible, provide the related medical diagnosis. He provides the sponsor with additional information concerning serious adverse events and must also respond to any request for additional information from the sponsor.

Any adverse event will be followed up by the investigator until it is completely resolved (stabilization at a level deemed acceptable by the investigator, or return to the previous state), even if the person has left the trial.

In both notifications and subsequent reports, research subjects are identified by a unique code number assigned by the sponsor.

- - 1. Assessing the severity and causality of serious adverse events related to the study

The investigator evaluates each adverse event collected during the course of the research:

1. its seriousness, in accordance with the definition given in Article R.1123-46 of the French Public Health Code (CSP), provided in Appendix 1;
2. the causal link between the serious adverse event and the clinical trial :

- Unrelated : no causal link with the research.
- Related : a causal link with the research is suspected. All adverse events for which the investigator or sponsor considers that a causal relationship with the clinical trial can be reasonably envisaged are considered suspected adverse events.
  - 1. Assessing the severity of the serious adverse events

The investigator assesses the severity of each serious adverse event using the following scale:

- Mild : tolerated by the subject, without interference with daily activities
- Moderate : sufficiently uncomfortable to affect daily activities
- Severe : prevents the subject from carrying out daily activities
  - 1. Deadlines for notifying the sponsor and means of transmission

The reporting period begins on the date the consent form is signed and extends throughout the participant's follow-up under the protocol. There is no time limit if the serious adverse event is likely to be related to the procedures or interventions added by the clinical trial.

The initial notification and, where applicable, any follow-up reports must be sent anonymously to the sponsor by e-mail, using a duly completed and validated notification form, to the following address:

**Biomedical Research Vigilance Unit, CHRU Tours**

**Mail: eig-uvrb@chu-tours.fr**

In the event of exceptional unavailability of e-mail, notifications may by default be sent by fax to the following number: 02 47 47 38 26.

The original notification form is kept at the investigator's site.

- - 1. Study-specific provisions
       1. Special situations

The following serious adverse events will not be reported in the observation booklet:

- Admission for social or administrative reasons;
- Hospitalization for medical or surgical treatment scheduled before the start of research participation;
- Hospitalization for a pre-existing pathology detected before the first research procedure, and which does not worsen;
- Hospitalization integrated into the experimental research plan and predefined by the trial protocol.

Adverse reactions associated with drug treatments unrelated to the research are reported by the investigator to the Centre Régional de Pharmacovigilance (Regional Pharmacovigilance Center).
Adverse reactions associated with ancillary medicinal products with marketing authorization used in the clinical trial are also reported by the investigator to the Centre Régional de Pharmacovigilance on which they depend.

The investigator should only unblind a clinical trial participant to treatment if it is relevant to the participant's safety. Blinding is maintained for all other individuals responsible for conducting the clinical trial and for those involved in data analysis and interpretation of results at the end of the clinical trial. Post-unblinding information is accessible only to those involved in assessing the safety of the clinical trial, including members of the independent monitoring committees.

- - 1. Notification of non-serious adverse events

All other non-serious adverse events will be reported on the « adverse event » form in the observation book, specifying the date of occurrence, description, intensity, duration, mode of resolution, etiology, imputability, and decisions taken.

- 1. The sponsor’s responsibilities

The sponsor is responsible for the ongoing evaluation of the safety of the clinical trial throughout its duration.

- - 1. Collection and evaluation adverse events

The sponsor records the reports received from the investigator and keeps a detailed register of all adverse events reported by the investigator.

- - - 1. Assessment of seriousness and causal relationship

Each adverse event reported by the investigator must be evaluated by the sponsor, including an assessment of its severity and of the causal relationship between the adverse event and the investigational drug, any ancillary treatments, or the research itself.

- - - 1. Assessment of the unexpected nature of adverse reactions

All serious adverse events for which the investigator and/or sponsor consider that a causal relationship with the research can reasonably be envisaged are considered suspected serious adverse events. The sponsor assesses the expected or unexpected nature of any suspected serious adverse event based on the reference safety information defined for the clinical trial: the Kalinox® Summary of Product Characteristics (SPC).

If an event is likely to be a suspected serious unexpected adverse reaction (SUSAR), the sponsor will unblind only the concerned participant. Information disclosed after unblinding is accessible only to those involved in assessing the safety of the clinical trial, including the independent monitoring committee. Blinding is maintained for all other personnel responsible for the conduct of the clinical trial and for those in charge of data analysis and result interpretation.

If, after unblinding, the event is confirmed to be a SUSAR, the reporting rules to the competent authorities apply.

- - 1. Safety reporting to authorities

Safety data means :

– Suspected serious and unexpected adverse reactions,
– Unexpected events affecting the benefit/risk ratio of the clinical trial,
– Serious safety violations: any violation likely to have a significant impact on the safety of a clinical trial subject,
– Urgent safety measures,
– Medication errors, non-compliant use, misuse, abuse.

The sponsor declares the safety data to the competent authorities from the date of authorization of the research in France, i.e., from the date on which the clinical trial authorization is obtained from the ANSM.

- - - 1. Immediate reporting
         1. Suspected unexpected serious adverse reactions

The sponsor shall report any suspected serious unexpected adverse reaction to the investigational medicinal product to the European Medicines Agency within the following timeframes:

- As soon as possible and no later than 7 days in the event of a suspected serious adverse reaction resulting in death or life-threatening condition;
- No later than 15 days from the date on which the sponsor becomes aware of it in all other cases.

The sponsor must report any additional relevant information concerning a suspected serious unexpected adverse reaction in the form of a follow-up report within 8 days of the initial notification. This 8-day timeframe applies to the first follow-up report.

If the sponsor receives significant new information regarding a previously reported suspected serious unexpected adverse reaction, the follow-up report including this new information must be submitted within a maximum of 15 days from the date the sponsor becomes aware of it.

- - - 1. Management and reporting of other safety data

The sponsor reports to the competent authorities via the European portal all unexpected events that may impact the benefit/risk ratio of the clinical trial but do not constitute suspected serious unexpected adverse reactions, without undue delay and no later than 15 days after becoming aware of them.

In the event of an unexpected event likely to seriously affect the benefit/risk ratio of the clinical trial, the sponsor and investigator must take appropriate urgent safety measures to protect participants. In such cases, the sponsor must notify the European Medicines Agency of the unexpected event and the urgent safety measures taken, without undue delay and no later than 7 days after the measures are implemented.

The sponsor must also notify the competent authorities of any serious breach at the time it occurs, without undue delay and no later than 7 days after becoming aware of it.

- - - 1. Annual safety report

Once a year, or upon request, the sponsor sends a safety report to the competent authorities via the European portal, taking into account all available safety information. The report is forwarded to the ANSM and the CPP for the entire duration of the clinical trial in France, i.e. until its end date in France (end of participation of the last person taking part in the clinical trial or, where applicable, the end date defined in the protocol). The annual safety report includes a list of all serious adverse events, a list of all suspected serious adverse reactions, and an analysis of this information with regard to the safety of research subjects.

The annual safety report is sent to the competent authorities within 60 days of the anniversary of the effective start of the research, corresponding to the date of initial authorization of the research by the ANSM.

- - 1. Information provided to investigators by the sponsor

The sponsor must inform all investigators concerned of any data that could have an adverse impact on the safety of research subjects, in particular any relevant information resulting from the analysis of suspected serious unexpected adverse reactions that could affect subject safety (either following receipt of an individual report or after review of cumulative data), as well as any new information that may impact the conduct of the clinical trial or the development program, including study suspension or a decision to amend the protocol for safety reasons.

- 1. Oversight committee

An Independent Monitoring Committee (IMC) will be responsible for advising the sponsor, in an advisory capacity, on the benefit/risk balance of the research and its conduct. To this end, it will examine all issues that may arise during the trial, particularly scientific, ethical, and safety concerns that could affect the risk/benefit ratio. Its independent members are appointed and mandated by the sponsor for the duration of the trial and are committed to active participation and to maintaining data confidentiality.

The operating procedures (e.g., frequency of meetings, data to be monitored) will be defined in the IMC Charter. The IMC will issue recommendations regarding the continuation of the trial (amendment, suspension, or termination), particularly in the event of serious adverse events that warrant a reassessment of the research’s risk/benefit ratio. The sponsor remains the sole decision-maker and, if necessary, will forward the IMC reports to the ANSM and the CPP.

- 1. Follow-up care methods and duration after adverse events

Each adverse event will be monitored until its complete resolution (stabilization at a level deemed acceptable by the investigator, or return to the previous state), even if the patient is withdrawn from the trial.

1. Regulatory and ethical considerations

The investigator undertakes to conduct this study in compliance with the applicable legislative and regulatory provisions governing research involving human subjects (European Regulation No. 536/2014 of April 16, 2014, on clinical trials). The investigator also commits to adhering to Good Clinical Practice and the World Medical Association’s Declaration of Helsinki.

- 1. CNIL

**The data collected during the trial will be processed electronically.**

This processing will be carried out in accordance with the provisions of the French Data Protection Act (Law No. 78-17 of January 6, 1978), as amended by Law No. 2018-493 of June 20, 2018, and with Regulation (EU) 2016/679 of the European Parliament and of the Council of April 27, 2016 (General Data Protection Regulation - GDPR), concerning the protection of individuals with regard to the processing of personal data and the free movement of such data.

Study participants may exercise their rights of access, rectification, erasure, restriction, and objection to the processing of their personal data by contacting the study coordinator or the sponsor, via the Data Protection Officer of CHRU de Tours (dpo@chu-tours.fr). For any complaints regarding the processing of health data, participants may also contact the French Data Protection Authority (Commission Nationale de l’Informatique et des Libertés - CNIL) at [https://www.cnil.fr](https://www.cnil.fr/).

This study falls under the “Reference Methodology” (MR-001). The CHRU de Tours, as the sponsor of the study, signed an undertaking to comply with this “Reference Methodology” on 12/02/2007.

- 1. Ethics Committee (Comité de Protection des Personnes)

The study protocol, information sheet, and informed consent form will be submitted to the Ethics Committee (Comité de Protection des Personnes – CPP) for approval.

Notification of the CPP's favorable opinion will be sent to the study sponsor and to the competent authority. An authorization request will also be submitted by the sponsor to the ANSM prior to the initiation of the study.

- 1. Substantial modifications

Any substantial modification to the protocol proposed by the investigator must be approved by the sponsor. Prior to implementation, the sponsor must obtain a favorable opinion from the Ethics Committee (CPP) and authorization from the ANSM, each within their respective areas of competence. If applicable, a new informed consent must be obtained from participants in the study.

- 1. Information and consent

Patients will be fully and clearly informed, in comprehensible terms, about the objectives and constraints of the study, the potential risks involved, the necessary monitoring and safety measures, and their right to refuse participation or to withdraw at any time without consequence.

All this information will be provided in an information and consent form given to the patient and, if applicable, to their legal representative in the case of guardianship or protective supervision.
The patient's free, informed, and written consent will be obtained by the investigator, or a delegated physician, prior to final inclusion in the study. Additional consent will be obtained from patients enrolled at CHRU de Tours for the collection and use of residual biological material from blood samples taken for vitamin B12 assays.

- 1. Biological sample collection

For patients included at CHRU de Tours, any residual biological samples not used for study-related analyses (vitamin B12 assay) will be stored in an open-ended biological collection maintained at CHRU de Tours. This collection, along with the associated data, may be used for future research related to the same scientific theme. The biological collection will be declared to the French Ministry of Higher Education and Research.

- 1. Insurance

The Sponsor shall, for the entire duration of the study, take out insurance covering its own civil liability as well as that of any physician involved in the conduct of the study, in accordance with Article L1121-10 of the French Public Health Code and European Regulation No. 536/2014. The Sponsor shall also ensure full compensation for any harm resulting from the research to the participant or their beneficiaries, unless it can demonstrate that the damage is not attributable to its own fault or that of any involved party. Compensation shall not be denied on the grounds of third-party actions or the voluntary withdrawal of a participant who initially consented to take part in the research.

- 1. Record-keeping

The study will be registered on an open-access website (ClinicalTrial) prior to inclusion of the ^1st^patient in the study. Registration will be updated regularly.

- 1. Archiving of documents and data at the end of the study

The following documents will be archived under the name of the study at the premises of the Cellule Promotion et Contrôle Qualité of the CHRU de Tours until the end of the operational retention period. These documents include :

- The study protocol and all appendices, including any amendments
- Individual data (authenticated copies of source documents)
- Follow-up documentation
- The final study report

At the end of the operational retention period, all documents to be archived—according to the CHRU de Tours procedure for “Filing and Archiving Documents Related to Biomedical Research”—will be transferred to the central archiving site (Service Central des Archives – Hôpital de Trousseau) and placed under the responsibility of the Sponsor, in accordance with institutional policies and for a duration compliant with applicable regulatory requirements, i.e., 25 years.

No document may be removed or destroyed without prior authorization from the Sponsor. At the end of the statutory retention period, the Sponsor will be consulted regarding destruction. All data, documents, and reports remain subject to audit or inspection.

1. Publication policy

14.1 General

Analysis of the study data will be performed by CIC INSERM 1415. The results of the statistical analyses will be compiled in a written report and forwarded to the study coordinator.

Any written or oral communication of the study results must be approved by the coordinating principal investigator and, where applicable, by the scientific committee established for the study.

- 1. Authorship

The publication of the main results shall acknowledge the sponsor, all investigators involved in patient recruitment and/or follow-up, the study methodologist, biostatistician, data manager, members of the study committee(s), and the funding source.

The recommendations of the International Committee of Medical Journal Editors (ICMJE), as outlined in the document "Recommendations for the Conduct, Reporting, Editing, and Publication of Scholarly Work in Medical Journals" (updated December 2015), will be followed.

- 1. Communication of results to study participants

In accordance with current regulations, participants will be informed of the overall results of the study at their request. A summary of the results will also be available on the European platform : https://euclinicaltrials.eu/search-for-clinical-trials.

- 1. Data sharing

Data collection and management will be carried out by the CHRU de Tours. The conditions governing the transfer of all or part of the database will be determined by the study sponsor and formalized in a written agreement.

1. Financial aspects
   1. Study budget

Additional study costs are covered by the Promoter.

- 1. Participant compensation

Research participants will be reimbursed for their travel expenses upon presentation of receipts, up to a maximum of €200 for the entire study (including all visits).

No compensation will be paid to participants.

Bibliography

Aizenstein HJ, Baskys A, Boldrini M, Butters MA, Diniz BS, Jaiswal MK, *et al* (2016). Vascular depression consensus report - a critical update. *BMC* Med **14**: 161.

Aizenstein HJ, Khalaf A, Walker SE, Andreescu C (2014). Magnetic resonance imaging predictors of treatment response in late-life depression. *J Geriatr Psychiatry* Neurol **27**: 24–32.

Amsterdam J van, Nabben T, Brink W van den (2015). Recreational nitrous oxide use: Prevalence and risks. *Regul Toxicol* Pharmacol **73**: 790–796.

Bakish D (2001). New standard of depression treatment: remission and full recovery. *J Clin Psychiatry* **62 Suppl 26**: 5–9.

Biogeau J, Desmidt T, Dujardin P-A, Ternifi R, Eudo C, Vierron E, *et al* (2017). Ultrasound Tissue Pulsatility Imaging Suggests Impairment in Global Brain Pulsatility and Small Vessels in Elderly Patients with Orthostatic Hypotension. *J Stroke Cerebrovasc* Dis **26**: 246–251.

Borm GF, Fransen J, Lemmens WAJG (2007). A simple sample size formula for analysis of covariance in randomized clinical trials. *J Clin* Epidemiol **60**: 1234–1238.

Chaki S (2017). Beyond ketamine: new approaches to the development of safer antidepressants. *Curr Neuropharmacol* doi:10.2174/1570159X15666170221101054.

Cipriani A, Furukawa TA, Salanti G, Geddes JR, Higgins JP, Churchill R, *et al* (2009). Comparative efficacy and acceptability of 12 new-generation antidepressants: a multiple-treatments meta-analysis. *Lance*t **373**: 746–758.

Dashdorj N, Corrie K, Napolitano A, Petersen E, Mahajan RP, Auer DP (2013). Effects of subanesthetic dose of nitrous oxide on cerebral blood flow and metabolism: a multimodal magnetic resonance imaging study in healthy volunteers. *Anesthesiolog*y **118**: 577–586.

Desmidt T, Brizard B, Dujardin P-A, Ternifi R, Réméniéras J-P, Patat F, *et al* (2017). Brain Tissue Pulsatility is Increased in Mid-Life Depression: A Comparative Study using Ultrasound Tissue Pulsatility Imaging. *Neuropsychopharmacology* doi:10.1038/npp.2017.113.

Desmidt T, Hachemi ME, Remenieras J-P, Lecomte P, Ferreira-Maldent N, Patat F, *et al* (2011). Ultrasound Brain Tissue Pulsatility is decreased in middle aged and elderly type 2 diabetic patients with depression. *Psychiatry* Res **193**: 63–64.

Desmidt T, Karim H, Andreescu C, Aizenstein H (To be Published). Cerebral physiological fluctuations in late-life depression: changes over a 12-week venlafaxine trial. *Neurobiology of Aging* .

Karim HT, Andreescu C, Tudorascu D, Smagula SF, Butters MA, Karp JF, *et al* (2017). Intrinsic functional connectivity in late-life depression: trajectories over the course of pharmacotherapy in remitters and non-remitters. *Mol* Psychiatry **22**: 450–457.

Kucewicz JC, Dunmire B, Giardino ND, Leotta DF, Paun M, Dager SR, *et al* (2008). Tissue Pulsatility Imaging of Cerebral Vasoreactivity During Hyperventilation. *Ultrasound in Medicine &* Biology **34**: 1200–1208.

Kucewicz JC, Dunmire B, Leotta DF, Panagiotides H, Paun M, Beach KW (2007). Functional Tissue Pulsatility Imaging of the Brain During Visual Stimulation. *Ultrasound in Medicine &* Biology **33**: 681–690.

Makedonov I, Black SE, Macintosh BJ. BOLD fMRI in the white matter as a marker of aging and small vessel disease. PLoS One. 2013;8(7):e67652. Published 2013 Jul 2. doi:10.1371

Makedonov I, Chen JJ, Masellis M, MacIntosh BJ, Alzheimer's Disease Neuroimaging Initiative (2016). Physiological fluctuations in white matter are increased in Alzheimer's disease and correlate with neuroimaging and cognitive biomarkers. *Neurobiol* Aging **37**: 12–18.

Nagele P, Duma A, Kopec M, Gebara MA, Parsoei A, Walker M, *et al* (2015). Nitrous Oxide for Treatment-Resistant Major Depression: A Proof-of-Concept Trial. *Biol* Psychiatry **78**: 10–18.

Pan A, Sun Q, Okereke OI, Rexrode KM, Hu FB (2011). Depression and risk of stroke morbidity and mortality: a meta-analysis and systematic review. *JAM*A **306**: 1241–1249.

Taragano FE, Bagnatti P, Allegri RF (2005). A double-blind, randomized clinical trial to assess the augmentation with nimodipine of antidepressant therapy in the treatment of "vascular depression." *Int* Psychogeriatr **17**: 487–498.

Taylor WD, Aizenstein HJ, Alexopoulos GS (2013). The vascular depression hypothesis: mechanisms linking vascular disease with depression. *Mol* Psychiatry **18**: 963–974.

Ternifi R, Cazals X, Desmidt T, Andersson F, Camus V, Cottier J-P, *et al* (2014). Ultrasound measurements of brain tissue pulsatility correlate with the volume of MRI white-matter hyperintensity. *J Cereb Blood Flow* Metab **34**: 942–944.

Vickers AJ (2003). How many repeated measures in repeated measures designs? Statistical issues for comparative trials. *BMC Med Res* Methodol **3**: 22.

Whiteford HA, Degenhardt L, Rehm J, Baxter AJ, Ferrari AJ, Erskine HE, *et al* (2013). Global burden of disease attributable to mental and substance use disorders: findings from the Global Burden of Disease Study 2010. *Lance*t **382**: 1575–1586.

Wen W, Sachdev P. [The topography of white matter hyperintensities on brain MRI in healthy 60- to 64-year-old individuals.](https://www.ncbi.nlm.nih.gov/pubmed/15110004)Neuroimage. 2004 May;22(1):144-54.

Zivin K, Yosef M, Miller EM, Valenstein M, Duffy S, Kales HC, *et al* (2015). Associations between depression and all-cause and cause-specific risk of death: a retrospective cohort study in the Veterans Health Administration. *J Psychosom* Res **78**: 324–331.

Appendix

Appendix 1. Definitions for safety assessment

In accordance with Article R1123-46 of the French Public Health Code and Article 2 of European Regulation N°536/2014, the following definitions apply:

- 1. **Experimental drug**

Drug tested or used as a reference, including as a placebo, in a clinical trial.

- 1. **Auxiliary medication**

Drug used for the purposes of a clinical trial in accordance with the protocol, but not as an investigational drug.

- 1. **Undesirable event**

Any harmful manifestation in a participant to whom a drug is administered, and which is not necessarily related to this treatment.

- 1. **Serious adverse event**

Any harmful event that, regardless of dose, requires hospitalization or prolongation of hospitalization, causes lasting or significant disability or incapacity, results in a congenital anomaly or malformation, endangers the life of the participant or results in death.

Certain medical events may endanger the subject or require intervention to prevent one of the above-mentioned characteristics/consequences. Such events, known as "significant medical events", are also considered serious adverse events.

- 1. **Adverse effect of an experimental drug**

Any noxious and unwanted reaction to an investigational drug at any dose.

- 1. **Serious unexpected adverse reaction**

Any serious adverse reaction whose nature, severity or course is not consistent with reference safety information.

- 1. **New fact**

Any new data that may lead to a reassessment of the risk-benefit ratio of the research or of the investigational product, to changes in the use of this product, in the conduct of the research, or in the documents relating to the research, or to the suspension, interruption or modification of the research protocol or similar research.

For trials involving the first administration or use of a health product in people with no medical condition: any new data that may lead to a reassessment of the benefit/risk ratio of the clinical trial or the drug being tested, to changes in the use of the drug, in the conduct of the clinical trial, or in the clinical trial documentation, or to the suspension, interruption or modification of the clinical trial protocol, and any serious adverse drug reactions.
