# Supplementary material for: Nitrous oxide for late-life depression with inadequate antidepressant response: a randomised controlled trial
Source: eClinicalMedicine. 2026 Apr 2;94:103860. doi: 10.1016/j.eclinm.2026.103860 (PMC13084366; doi:10.1016/j.eclinm.2026.103860)
Supplement: Statistical Analysis Plan [file mmc3.docx]

| 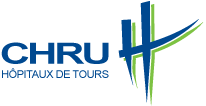 | **Statistical Analysis Plan** |
| --- | --- |

Nitrous oxide in Treatment-Resistant Depression in the Elderly : a randomized double-blinde comparator-controlled trial : PROTOBRAIN

***Supplement 2: Statistical Analysis Plan***

***22 may 2025***

**Pr Thomas DESMIDT**

CPU-CMRR, CHRU Tours

**Document produced by:** Dr Pierre Poupin

SUMMARY

I. Rationale 4

II. Objectives 4

1. Primary objective 4

2. Secondary objective 4

III. Study methodology 5

1. Study design 5

2. Population 5

3. Outcome measures 6

a) Baseline patient’s characteristics 6

b) Adherence to the intervention 7

c) Outcome measures related to the primary objective 7

d) Subgroups 7

e) Outcome measures related to the secondary objective 7

4. Study procedures 8

5. Number of subjects to include 8

IV. Statistical Analysis Strategy 9

1. Data Analysis – General principles 9

a) Missing data 9

b) Alpha risk 9

c) Analysis population(s) 9

2. Baseline patient’s description 9

3. Primary analysis 9

4. Subgroups analysis 10

5. Secondary analysis 10

6. Modifications to the analysis strategies compared to the protocol 10

7. Implementation 10

1. Rationale

Depression is a severe disorder not only because of its high prevalence but also due to the limited response rate to conventional antidepressants targeting the aminergic system. Older adults are particularly poor responders to standard antidepressant treatments. It has been demonstrated that impaired cerebral vascular function—whose prevalence increases with age—is one of the main factors contributing to the reduced response to antidepressants in the elderly.
Recently, agents with NMDA receptor antagonist properties, particularly ketamine, have shown efficacy in the treatment of treatment-resistant depression. However, the risk-benefit balance of this treatment is questionable due to the frequency and nature of side effects (notably, the emergence of psychotic symptoms in more than one-third of patients). Another NMDA receptor antagonist, Nitrous Oxide (N₂O, also known as "laughing gas" and commonly used in anesthesia), has recently demonstrated antidepressant effects in a group of patients with treatment-resistant depression, with satisfactory tolerability. Nevertheless, to date, no study has evaluated the efficacy and safety of N₂O in late-life depression.

Given that cerebrovascular factors are involved in depression in the elderly, and that N₂O has significant effects on cerebrovascular reactivity—reflecting a global cerebral response—we hypothesize that N₂O will be particularly effective in late-life depression and that cerebrovascular changes induced by N₂O exposure will be more pronounced in patients who exhibit a significant antidepressant response.

We propose a double-blind study comparing the efficacy of Nitrous Oxide (N₂O) as an add-on to a conventional antidepressant treatment versus a conventional antidepressant treatment combined with medical air, on the progression of depressive symptoms in a population of elderly patients with treatment-resistant depression. We also aim to assess the impact of N₂O treatment not only clinically but also on cerebrovascular function using innovative brain imaging techniques (MRI and ultrasound).

1. Objectives
2. Primary objective

To compare changes in depressive symptomatology at 2 hours, 24 hours, Week 1, and Week 2 following exposure to N₂O versus medical air in a population of elderly subjects with a major depressive episode meeting criteria for treatment-resistant depression and receiving antidepressant treatment.

1. Secondary objective
2. To compare the dynamics of changes in Cerebral Pulsatility as measured by Ultrasound (Tissue Pulsatility Imaging – TPI) during gas diffusion between responder patients (MADRS decrease > 50%), non-responders (MADRS decrease < 50%), and patients in the comparator group.
3. To compare structural (brain volumes, cerebral leukoencephalopathy) and functional (ASL perfusion, BOLD-based Cerebral Pulsatility and Connectivity) MRI data at baseline between responder patients, non-responders, and patients in the comparator group.
4. To compare changes in depressive symptomatology between subjects in the N₂O group versus the comparator group, as measured by the Hamilton Depression Rating Scale (HDRS) for depressive symptom severity, the Clinical Global Impression (CGI) scale for overall clinical improvement, and the QIDS-SR for self-rated mood.
5. To compare tolerability between subjects in the N₂O group versus the comparator group, as assessed by the SSI scale for suicidal ideation, the YMRS for manic symptoms, and the CADSS and BPRS for dissociative symptoms.

Only the analyses of the secondary objectives 3 and 4 will be detailed. The elements related to objectives 1 and 2 will therefore not be mentioned in the remainder of this document.

1. Study methodology
2. Study design

Superiority clinical trial, multicenter, controlled, double-blind for both participants and investigators, as an add-on to antidepressant treatment, with stratified randomization by center in a 1:1 ratio between:

- Inhalation of N₂O for 1 hour
- Inhalation of medical air for 1 hour

1. Population

Patients over 60 years old meeting the criteria for a major depressive episode resistant to at least one adequately administered antidepressant, as assessed by the MGH-ATRQ scale.

***Inclusion criteria***

- Age between 60 and 90 years
- Diagnosis of a major depressive episode according to DSM-5 criteria, confirmed by the Mini International Neuropsychiatric Interview (MINI)
- MADRS depression score greater than 20 (Montgomery Åsberg Depression Rating Scale)
- Patients resistant to at least one adequately administered antidepressant for the current depressive episode, as assessed by the MGH-ATRQ scale
- Patients able to undergo N₂O administration via a facial mask
- Signed informed consent
- Affiliated with a social security system or equivalent coverage

***Non-inclusion criteria***

- Bipolar disorder, schizophrenia, or documented neurodegenerative disease as assessed by the MINI and MMSE (non-inclusion if MMSE < 24/30); substance use disorder
- Unstable somatic condition (particularly neurological or cardiac conditions likely to interfere with N₂O administration)
- Presence of active and significant psychotic symptoms, at the investigator’s discretion
- Contraindications to the use of N₂O: pneumothorax, emphysema, intestinal obstruction, intracranial hypertension, known and untreated vitamin B12 or B9 deficiency
- Contraindications to MRI, including claustrophobia
- Legal incapacity and/or any other circumstance rendering the patient unable to understand the nature, purpose, or consequences of the study
- Participation in another drug clinical trial or currently within an exclusion period due to prior participation in a clinical trial

1. Outcome measures
2. Baseline patient’s characteristics

Baseline characteristics of the patients:

- Age
- Sex
- Body Mass Index
- Systolic and diastolic blood pressure
- Maudsley Staging Method score
- Number of previous depressive episodes
- Duration of the current depressive episode
- CIRS: Cumulative Illness Rating Scale
- Current antidepressant treatment
- Other psychotropic treatments
- MADRS score
- MMSE score
- HDRS: Hamilton Depression Rating Scale
- CGI score: Clinical Global Impression
- QIDS-SR score: Quick Inventory of Depressive Symptomatology – Self Report
- STAI-Y-A score: State-Trait Anxiety Inventory
- VAS score: Visual Analogue Scale
- SSI score: Scale for Suicidal Ideation

1. Adherence to the intervention

This is a single hetero-administration performed by a healthcare professional, with documentation of the start and end times of the administration of either N₂O or medical air.

1. Outcome measures related to the primary objective

The primary outcome measure is the MADRS score, a discrete quantitative psychometric scale with a total score ranging from 0 to 60. It will be assessed at baseline and then measured at 2 hours, 24 hours, 7 days, and 14 days post-exposure.

1. Subgroups

Any subgroup analysis is planned.

1. Outcome measures related to the secondary objective

Complementary scales to MADRS for monitoring depressive symptom evolution:

- The 17-item Hamilton Depression Rating Scale
- The CGI: Clinical Global Impression
- The QIDS-SR: Quick Inventory of Depressive Symptomatology – Self Report
- The STAI-Y-A: State-Trait Anxiety Inventory
- The VAS: Visual Analogue Scale

Scales for assessing potential adverse effects:

- The SSI: Scale for Suicidal Ideation
- The YMRS: Young Mania Rating Scale
- The CADSS: Clinician-Administered Dissociative States Scale
- The BPRS: Brief Psychiatric Rating Scale

Collection of adverse events/effects.

1. Study procedures

Inclusion visit with clinical and psychometric assessment: demographic data, medical and surgical history summarized by the Cumulative Illness Rating Scale (CIRS), level of resistance of the depressive episode (measured by the Maudsley Staging Method), treatments, and psychometric scales including MADRS and all scales assessing depressive symptoms (Hamilton – for depressive symptom severity; CGI – for overall clinical impression; QIDS-SR – for self-assessed mood) as well as adverse effects (SSI – for suicidal ideation; YMRS – for manic symptoms; CADSS – for dissociative symptoms; BPRS – for psychotic symptoms).

Randomization to N₂O or medical air.

Follow-up visits scheduled at 2 hours post-exposure, at 24 hours (Day 1), 7 days (Week 1), and 14 days (Week 2), with collection of: MADRS and all scales related to secondary outcome measures (Hamilton – for depressive symptom severity; CGI – for overall clinical impression; QIDS-SR – for self-assessed mood) and adverse events (SSI – for suicidal ideation; YMRS – for manic symptoms; CADSS – for dissociative symptoms; BPRS – for psychotic symptoms).

1. Number of subjects to include

Based on the literature (Nagele *et al*, 2015), the following assumptions can be made:

- Expected mean change of –5.5 points in the N₂O group
- Expected mean change of –2.8 points in the comparator group
- Standard deviation of 5 points

This would require the inclusion of 73 patients per group to achieve 90% power with a 5% alpha risk. However, considering the presence of a baseline measurement and four follow-up measurements (2h, 24h, Week 1, and Week 2), and assuming a correlation of 0.5 between baseline and follow-up measurements (a low correlation, conservative assumption), the required sample size is 28 patients per group, based on the approach by Borm and Vickers (Borm et al., 2007; Vickers, 2003). This number was rounded to 30 per group (15 per centers as the number of participating centers initially planned was 4).

1. Statistical Analysis Strategy
2. Data Analysis – General principles
3. Missing data

For the primary outcome, no imputation strategy is planned for missing data. It should be noted that the use of a mixed-effects model allows taking into account all the available measurements for a subject and implicitly “imputes” missing outcome data with no bias under the assumption of data missing not at random (MAR).

1. Alpha risk

The statistical significance threshold is set at 5% (two-sided), and 95% two-sided confidence intervals will be calculated for all estimates.

1. Analysis population(s)

All randomized subjects, except those who later object to the use of their data, are included in the analysis according to the group to which they were randomized, regardless of subsequent events.

1. Baseline patient’s description

The number of participants per group at each stage of the trial, along with the reasons for study withdrawal or missed follow-up visits, will be documented in a flow diagram, in accordance with CONSORT guidelines.

All baseline data will be summarized by treatment group using the mean and standard deviation for normally distributed quantitative variables, the median and interquartile range for other quantitative variables, and counts and percentages for qualitative variables. No statistical tests will be performed.

1. Primary analysis

The analysis of the primary outcome will be based on a linear mixed-effects regression model to account for the correlation between repeated measurements within the same subject. The model will include a fixed effect for time (treated as a categorical variable), a fixed effect for the randomization group (N₂O versus medical air), a fixed effect for the interaction between randomization group and time, and a random intercept for subject. This random subject intercept will model the correlation between repeated measurements within the same subject. Parameters estimation for the linear mixed-effects regression model will be performed using the Restricted Maximum Likelihood (REML) method, based on the lmer function from the lme4 package in R. The p-value associated with the interaction between group and the final time point (Week 2) will be used to draw conclusions regarding the primary analysis of the trial. The point estimate of the interaction term will correspond to the between-group difference at Week 2. This estimate will be reported along with its 95% confidence interval.

1. Subgroups analysis

Any subgroup analysis is planned

1. Secondary analysis

The models used for the analysis of secondary outcome measures will be similar to the one used for the primary outcome, except for the CGI-Improvement scale. For this scale, responses will be grouped into two categories: a score of 1 or 2 will corresponds to the very much better or a much better category while a score of 3 or higher will corresponds to the minimally better to very much worse category. Intergroup comparisons at each time point will be performed using a χ² test or a Fisher exact test depending on whether the validity conditions are met or not.

It should be noted that the analyses of the secondary outcomes are exploratory in nature, and no correction for alpha risk inflation is planned.

1. Modifications to the analysis strategies compared to the protocol

A modification concerns the analysis of a secondary outcome: the CGI-Improvement. The CGI score was not analyzed using a linear mixed regression model as mentioned in the protocol. The individual scores were dichotomized in 2 categories (as described previously) and comparisons between groups at each time of measurements were performed using a χ² test or a Fisher exact test depending on whether the validity conditions are met or not.

1. Implementation

Statistician : Pierre Poupin, Clinical Investigation Center INSERM 1415, University Hospital Center of Tours.

Software : SAS version 9.4, R version 4.1.2.
